# Generation of iPSC-derived limb progenitor-like cells for stimulating phalange regeneration in the adult mouse

**DOI:** 10.1038/celldisc.2017.46

**Published:** 2017-12-19

**Authors:** Ying Chen, Hanqian Xu, Gufa Lin

**Affiliations:** 1 Department of Genetics Cell Biology and Development, Stem Cell Institute, University of Minnesota, Minneapolis, MN, USA; 2 Research Centre for Translational Medicine, Shanghai East Hospital, School of Life Sciences and Technology, Tongji University, Shanghai, China

**Keywords:** adult, phalange regeneration, limb progenitor cells, fibrin body, transplantation

## Abstract

The capacity of digit tip regeneration observed both in rodents and humans establishes a foundation for promoting robust regeneration in mammals. However, stimulating regeneration at more proximal levels, such as the middle phalanges (P2) of the adult mouse, remains challenging. Having shown the effectiveness of transplantation of limb progenitor cells in stimulating limb regeneration in *Xenopus*, we are now applying the cell transplantation approach to the adult mouse. Here we report that both embryonic and induced pluripotent stem cell (iPSC)-derived limb progenitor-like cells can promote adult mouse P2 regeneration. We have established a simple and efficient protocol for deriving limb progenitor-like cells from mouse iPSCs. iPSCs are cultured as three-dimensional fibrin bodies, followed by treatment with combinations of Fgf8, CHIR99021, Purmorphamine and SB43542 during differentiation. These iPSC-derived limb progenitor-like cells resemble embryonic limb mesenchyme cells in their expression of limb-related genes. After transplantation, the limb progenitor-like cells can promote adult mouse P2 regeneration, as embryonic limb bud cells do. Our results provide a basis for further developing progenitor cell-based approaches for improving regeneration in the adult mouse limbs.

## Introduction

Although mammals, including humans, have lost the capacity to regenerate injured limbs, the prospect of human limb regeneration is encouraged by the observation that the extreme distal end of human fingertip can regenerate. When treated conservatively, injury in the distal part of the terminal phalange (P3) can result in a regeneration response that restores the fingertip. But finger injuries proximal to the nail bed lead to a wound-healing response and scar formation, with no regeneration [1–4]. These observations of human digit tip regeneration suggest that humans retain a limited ability to regenerate, and establish the foundation on which we can explore ways to stimulate more dramatic regeneration in human limbs.

The mouse digit has become a good mammalian model for regeneration studies because its regeneration characteristics resemble that of the human. It also has level-dependent responses after amputation [5–8]. The mouse can regenerate up to 40% of the distal part of the terminal phalange (P3). However, the P3 will not regenerate in amputations involving over 75% of its length. And there is no regeneration at all when the amputation level is through the middle phalange (P2) or at levels more proximal. This regeneration response is the same in both neonatal and adult mice [6, 8].

In recent years, there have been substantial advances in the understanding of the cellular and molecular mechanisms of mouse digit tip regeneration [9–12] and there have been some attempts to stimulate proximal digit regeneration [13–16]. Most of the treatments involved the local application of proteins either relevant to extracellular matrix remodeling or to promote specific digit tissue type outgrowth. The underlying rationale for these methods is that the exogenous factors can potentially activate or recruit resident progenitor cells, which would otherwise not be available at the site of injury. However, the outcomes of these methods vary a lot and generally were not satisfactory. This is probably because the proliferation, migration and differentiation potential of the resident cells are not equivalent to those of the regeneration-competent progenitor cells in the distal digit tips.

An alternative approach to stimulating regeneration is supplementation of cells that resemble the regeneration-competent progenitor cells. It is now accepted that in both amphibian and mammalian models, limb and digit regeneration is mediated through the contribution of multiple progenitor cell types [9, 11, 17]. In our previous work, we promoted limb regeneration in the forelimbs of the non-regenerating frog *Xenopus laevis* through a cell transplantation-based approach [18]. The key of this approach is using embryonic limb progenitor cells. We now propose the cell transplantation-based approach as an alternative method to promote regeneration of the proximal mouse phalanges.

Here we report that with an optimized transplantation procedure to the middle phalange of digit 3 (digit 3 phalange 2 (D3P2)), embryonic limb cells supplemented with growth factors survive for a long time and can stimulate bone and soft tissue outgrowth. In addition, we establish a protocol for generating limb progenitor cells by utilizing an induced pluripotent stem cell (iPSC) (*Prx1Cre:mT/mG*) line. Using green fluorescent protein (GFP) expression as an indication of limb progenitor-like cell status, we identified a combination of growth factors and small molecules for inducing bud-like structures from the iPSCs cultured as three-dimensional fibrin bodies (FBs). Like the embryonic limb cells, these iPSC-derived limb progenitor-like cells can also stimulate phalange regrowth in the adult P2 amputation. Thus, our findings provide good evidence for the effectiveness of using progenitor cell transplantation to provoke regeneration in the adult mouse.

## Results

### Optimized conditions for limb progenitor cell transplantation into adult mouse P2 stumps

**Figure 1:**
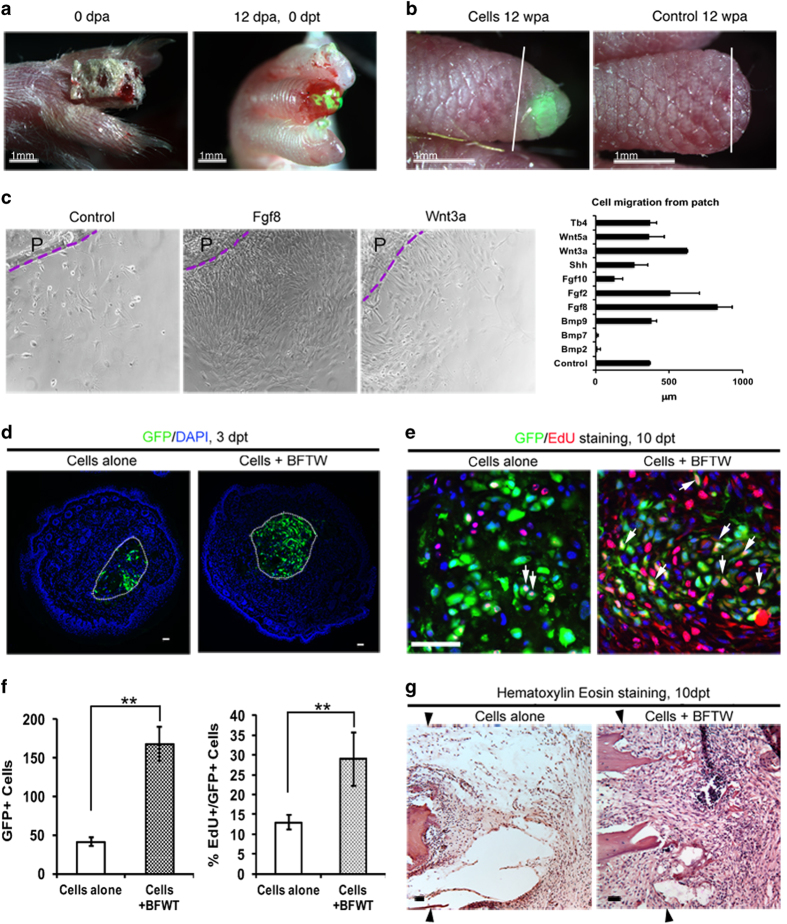
Optimization of cell transplantation in adult mouse D3P2 stump. (**a**) An amputated P2 digit was covered with a Hyphecan cap immediately after amputation to facilitate healing, and cell transplantation was performed at 12 dpa, by placing cells in fibrin gel under the re-opened wound epithelium. (**b**) Long-term retention of transplanted cells, observed at 12 weeks post amputation (wpa), with an elongated P2, in contrast to the blunt stump in control P2 amputations. White lines in (**b**) indicate amputation levels. Scale bars: 1 mm. (**c**–**g**) Selection of growth factors for cell transplantation in adult D3P2. (**c**) Distance of cells migrating out from fibrin patch in the presence of growth factors, measured in 10-day cultures. (**d**) Cross-sections of D3P2 stumps (3 dpt) with cell transplantation alone, or transplanted with cells and growth factor combination BFTW (Bmp2+Fgf8+Thymosin b4+ Wnt3a). The cell transplant is outlined with white dotted lines. (**e**) Analysis of cell proliferation in D3P2 stumps, at 10 dpt. White arrows indicate examples of EdU+ GFP+ cells. (**f**) Statistical analysis of GFP+ cells, and EdU+/GFP+ (proliferating) cells in the transplants, **P<0.01, Student’s *t*-test, *n*=3. (**g**) Hematoxylin and eosin staining on parasagittal sections of D3P2 after transplantation, at 10 dpt, showing larger cell mass in the P2 after transplantation of cells together with growth factors. Black arrowheads indicate amputation levels.

An earlier study showed the regeneration potency of embryonic limb buds upon transplantation onto neonatal mouse limbs [19]. However, in very young mice rapid growth and significant hypertrophy after limb and digit amputation render the effort of regeneration stimulation difficult to analyze. Thus, we used adult mice (8 weeks of age and older) to establish measurements for stimulating regeneration unambiguously, as there is little hypertrophy of the amputated bones in the adult mouse. Because of the constant scratching behavior of the mouse, it is hard to retain grafted cells at the P2 stump. We tested several methods and found that the cells were best retained when the cell-containing fibrin matrix was transplanted 1–2 days after the wound scab had fallen off (10–12 days post amputation, dpa). To facilitate healing, we covered the P2 stump with a Hyphecan cap. This is a dressing material made from natural sources (such as crab shells) and has been used to treat skin burns in human patients [3]. Around 12 dpa, we prepared the embryonic limb progenitor cells in fibrin gel and inserted the graft underneath the wound epithelium. Then, we placed the cell graft near the amputated bone stump (Figure 1a). With this approach, we found that transplanted cells survived a long term, and enabled the formation of a longer phalange than in the un-grafted control cases (Figure 1b).

Our previous work showed that exogenous factors are important to enable the transplanted progenitor cells to improve limb regeneration. To identify factors required for limb progenitor cells in the mouse P2 stump, we examined various growth factor combination(s) for maintaining both the proliferation and differentiation potential of embryonic limb progenitor cells. Based on the signaling pathways involved in early limb development and regeneration, we first tested a panel of growth factors by *in vitro* cell culture analysis. These included Activin A, Bmp2, Bmp4, Bmp7, Bmp9, Fgf2, Fgf8, Fgf10, Shh, Wnt3a, Wnt5a, Tβ4 (thymosin beta 4) and small molecules including CHIR99021, BIO, and Purmorphamine. We found that Bmp2, Fgf8 and Wnt3a have the most promoting effect on cell proliferation (Supplementary Figure S1a). Then, we performed further selection by grouping the growth factors. We identified that combinations of BF (Bmp2+Fgf8), BFT (Bmp2+Fgf8+Tβ4) and BFW (Bmp2+Fgf8+Wnt3a) have the most significant effect on stimulating cell proliferation *in vitro* (Supplementary Figure S1a).

We also analyzed the ability of selected growth factors in promoting the differentiation potential of limb progenitor cells toward chondrocytes and osteoblasts. We cultured the isolated limb progenitor cells under osteoblast/chondrocyte induction conditions and performed immunohistochemistry and real-time RT-PCR analysis for *Col2a1, Sox9, Runx2, Osteopontin (OPN)* and *Osteocalcin (OC)* (Supplementary Figure S1b). The results showed that combinations of BW (Bmp2+Wnt3a), BF, BT (Bmp2+Tβ4) are good candidates for stimulating bone differentiation. Combinations of BFT, BFW and BFTW (Bmp2+Fgf8 +Tβ4+Wnt3a) are the most promising for promoting differentiation of the limb progenitor cells toward the cartilage lineage (Supplementary Figure S1c).

We further examined the effect of growth factors on migration of the limb progenitor cells, as the cells are transplanted in a fibrin matrix to the amputated P2. Both Fgf8 and Wnt3a can stimulate cell migration out of fibrin gel patches (Figure 1c). These *in vitro* results prompted us to further test the combination BFTW in the cell transplantation studies.

We transplanted limb progenitor cells isolated from *nucGFP* transgenic embryonic limb bud mesenchyme into athymic nude mice P2 stumps, and analyzed the survival and proliferation of the transplanted cells. At 3 days post transplantation (dpt), we found more GFP cells in the transplants supplemented with BFTW (cells+BFTW) than that in the transplants with cells alone (Figure 1d and f). This shows that the application of BFTW factors supported the survival of the transplanted cells. We analyzed whether these factors also promote proliferation. Indeed, at 10 dpt, we observed much more proliferation in the cells+BFTW transplants (Figure 1e and f). Consequently, we observed a greater mass of cells accumulated in the stumps of cells+BFTW groups than in the stumps transplanted with cells alone (Figure 1g).

### Embryonic limb progenitors promote adult mouse P2 regeneration

**Figure 2:**
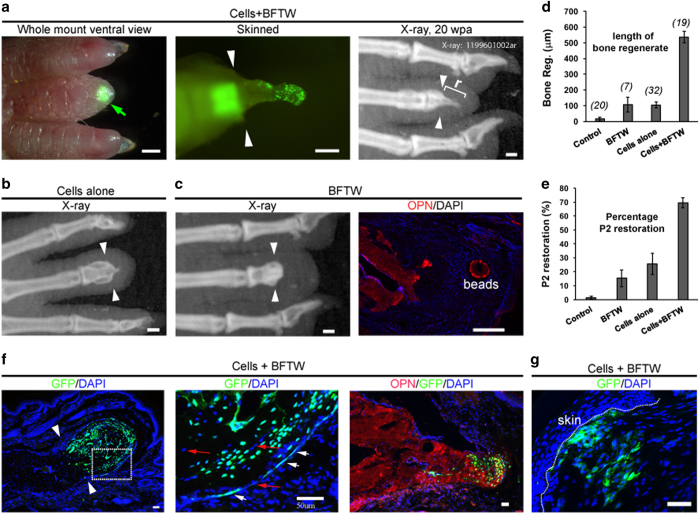
D3P2 regeneration after transplantation of embryonic limb progenitor cells together with growth factors. (**a**) Examples of adult mouse D3P2 after *nGFP* embryonic limb progenitor cell transplantation (with BFTW factors), under fluorescent microscope, after skin and soft tissue removal, and by X-ray imaging. GFP+ cells are in the bone regenerate. The green square area is auto fluorescence. X-ray image obtained at 20 weeks post amputation (wpa) is shown. Arrowheads indicate amputation levels. r indicates the regenerated bone. (**b**) Example of D3P2 transplanted with limb progenitor cells alone. (**c**) Example of non-regenerating bone in D3P2 implanted with BFTW beads only. Minimal regenerated bone can be detected with OPN (red). (**d**) Regeneration of bone as measured on X-ray images (determined as *r* in **d**). Error bars: standard error. Sizes of samples are shown in parenthesis*. P*<0.01, analysis of variance (ANOVA; one way) analysis. (**e**) Restoration of D3P2 determined by *r*/(length of amputated P2)×100%. (**f**–**h**) Distribution of transplanted limb progenitor cells in the P2 regenerates. (**f**) The regenerated bone contains both GFP+ and GFP− cells. White arrows indicate green cells at the outside surface of the regenerated bone. Red arrows indicate examples of host cells. OPN marks the regenerated bone. (**g**) GFP+ cell clusters found in the loose connective tissues in the distal stumps, underneath the skin (outlined with dotted line). White arrowheads indicate amputation levels. Scale bars in (**a,** **b**): 0.2 mm; (**f**, **g**): 50 μm.

Based on the above analysis, we transplanted embryonic limb progenitor cells provided with combinations of growth factors absorbed onto Affi-Gel blue beads, and analyzed the P2 regeneration by X-ray imaging and skeletal preparations. By fluorescence microscopy and X-ray imaging, we found that the combination of cells+BFTW could significantly promote regeneration after D3P2 amputation (Figure 2a). Although all stimulated bone regrowth was in a tapered shape, it did integrate nicely into the P2 stump (Figure 2a and f). By X-ray imaging, we observed that the bone regenerate continued to increase in size. All control animals failed to regenerate their phalanges (*n*=20), with negligible regrowth of bones and little regeneration of the soft tissues. Transplantation of limb progenitor cells alone (*n*=32), or implantation of beads containing growth factors alone (BFTW, *n*=7), only minimally improved bone outgrowth (Figure 2b and c). From the X-ray images, we measured the length of bone regeneration (Figure 2d), and calculated the percentage of final D3P2 length restored (Figure 2e). The P2 lengths in digits 2–4 of adult mice changed only slightly between 20 and 49 weeks of age (<5% increase). We determined that the average length of D3P2 is 1.6 mm before amputation (Supplementary Table S1). We measured the length of the D3P2 stumps, and calculated the length of D3P2 needed to be regenerated, which is about 0.8 mm on average. Therefore, in the group of transplanted cells+BFTW, a 0.55 mm regeneration corresponded to 70% restoration of the D3P2 (Figure 2e). This analysis confirmed the efficacy of grafts of limb progenitor cells plus growth factors for promoting bone regeneration.

The expression of GFP in the transplanted cells allowed us to track the behavior of the implanted cells in the regenerates. The GFP+ donor cells contributed mainly to the regenerated skeletal structure, and integrated nicely into the bone stumps (Figure 2f). GFP+ cells were found both within the bone and on its outside surface (elongated cells, short white arrows). The regenerated bone also contained GFP− cells (red arrows), indicating that host cells were recruited into the regenerate. By counting GFP+ cells, we calculated that about 45% (±7%) of the cells in the regenerated bone (marked by OPN shown in red, Figure 2f) were from the transplanted cells (*n*=4). The connective tissues were mainly host-derived (GFP−), but in two specimens we also found clusters of GFP+ cells in the soft tissues under the distal skin (Figure 2g). Thus, the cell labeling clearly shows that both the implanted limb progenitor cells and the host cells contribute to the regeneration of the bone and the connective tissues.

### Generation and characterization of limb progenitor-like cells from Prx1Cre:mT/mG mouse iPSCs

Our above results confirmed that embryonic limb progenitor cells with suitable growth factors can promote phalange regeneration in the adult mouse. However, any potential application of this technique to humans would require collecting large amounts of cells from embryonic limb buds and this would raise considerable ethical concerns. By contrast, iPSCs can be obtained in unlimited quantity and the technology of deriving cells of interest from them has great promise in cell-based therapies [20, 21]. To generate progenitor cells suitable for transplantation into the P2 stump, we established murine iPS cell lines from *Prx1Cre:mT/mG* embryonic fibroblasts (Supplementary Figure S2). The *mT/mG* transgenic cells express membrane-targeted tandem dimer Tomato (mT) before Cre recombination, and switch on membrane-targeted GFP (mG) after Cre recombination [22]. The mouse *Prx1* promoter drives Cre recombinase specifically in the developing limb mesenchyme [23] and *Prx1* expression has previously been associated with regeneration competence [24]. So iPS cells generated from tail fibroblasts express tdTomato (2c *Prx1Cre:mT/mG* miPSC), whereas iPS cells generated from embryonic limb mesenchymal cells express GFP. In our experiments, we used the cell line derived from tail fibroblasts (2c iPSC). We reasoned that these cells could be used to determine conditions for deriving limb progenitor cells, as indicated by the switching of red fluorescence to green fluorescence when *Prx1* becomes activated.

**Figure 3:**
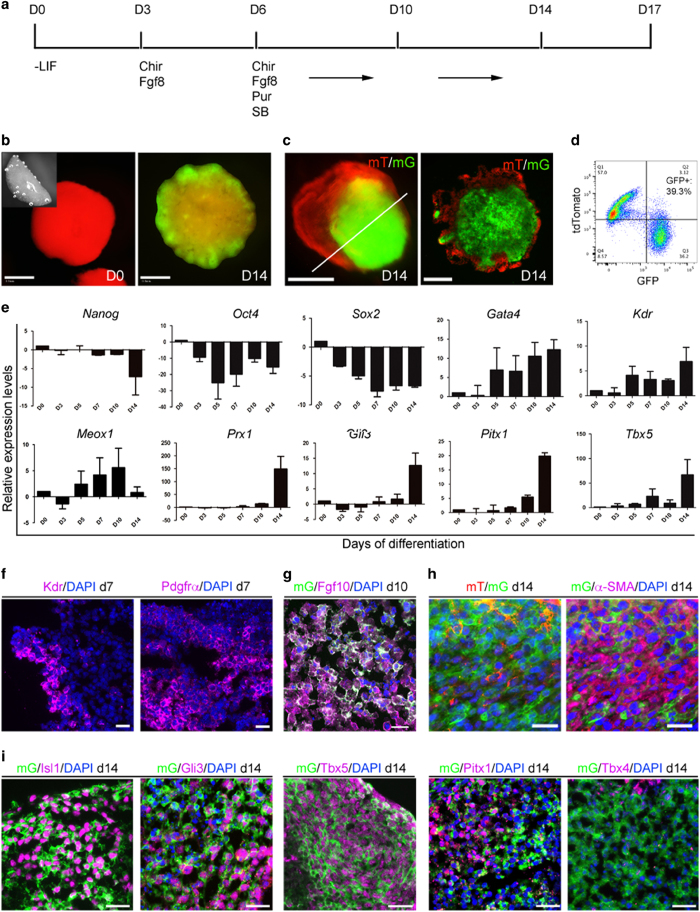
Derivation of limb progenitor-like cells from 3D FB culture of *Prx1Cre:mT/mG* iPSCs. (**a**) Diagram of the protocol used in this study. LIF (leukemia inhibitory factor), Chir (CHIR99021, 3 μM), Fgf8 (10 ng/ml), Pur (Purmorphamine, 4 μM), SB (SB431542, 2 μM). (**b**) Examples of FBs freshly made and cultured for 14 days (d14). Inset in d0 is a side view of the hemispherical FB immediately after transfer to culture medium. Multiple budding structures are GFP+ in a d14 FB (**b**). (**c**) A smaller bud-like structure, with a GFP+ core covered by tdTomato+ outside layer, revealed in cross-section. (**d**) Flow cytometry analysis of GFP+ cells in D14 FBs, showing about 40% cells were induced to switch on GFP expression. (**e**) Real-time PCR detection of gene expression in FBs, for pluripotency markers (*Nanog, Oct4, Sox2*), lateral plate mesoderm markers (*Gata4, Kdr, Meox1)* and limb field related genes *(Prx1, Gli3, Pitx1, Tbx5*). Gene expression levels were normalized to glyceraldehyde 3-phosphate dehydrogenase (GAPDH), and compared with d0 specimens. Results were from three independent experiments. (**f**) Detection of mesoderm markers Kdr and Pdgfrα at d7 of differentiation. (**g**) Detection of Fgf10 in day 10 FB cultures. (**h**) Induced GFP+ cells expressed mesodermal marker α-SMA (in purple), detected by immunofluorescence. (**i**) Detection of Isl1, Gli3, Tbx5, Pitx1 and Tbx4 in induced GFP+ cells by immunofluorescence. Scale bars: (**b**): 0.5 mm, (**c**): 200 μm, (**f**–**i**): 20 μm.

Using this 2c iPS cell line, we set out to establish a protocol for generating limb progenitor-like cells. Our initial effort focused on selecting growth factors and culturing of the 2c iPS cells in a three-dimensional fibrin matrix (which we call a FB). We identified Activin A, Wnt3a, Fgf8 and Shh as candidates for inducing limb progenitor cells, by following GFP expression in the FB cultures. Then, we tested various small molecules to minimize the need for protein growth factors. These trials led to the simple protocol shown in Figure 3a. This involves culturing FBs first with the Gsk3 inhibitor CHIR99021 and Fgf8, and then with the addition of the Shh agonist Purmorphamine, and the TGFβ type I receptor inhibitor SB431542.

During the 2-week differentiation in this protocol, the hemispherical FBs grew with an average of 20% increase in the diameter and form multiple bud-like structures (Figure 3b). The bud-like structures sometimes separated from the FB, and often contain a green mesenchyme core covered by an outer layer of red cells (Figure 3c). Flow cytometry analysis showed that about 40% of the cells in the buds were GFP+ (Figure 3d). We used real-time PCR analysis to detect gene expression changes during the differentiation process. After leukemia inhibitory factor withdrawal, expression of the pluripotency markers (*Nanog, Oct4, Sox2*) was downregulated. Expression of lateral plate mesoderm markers *Gata4*, *Kdr, Meox1* [25–27] was induced in the FBs between day 5 and day 7. In d14 FBs, limb field-related genes *Prx1, Pitx1, Tbx5* [28, 29] were also activated (Figure 3e).

We used immunofluorescence analysis to better characterize the bud-like FB cultures. Consistent with the PCR results, Kinase insert domain receptor (Kdr) and Platelet derived growth factor (PDGF) receptor alpha (Pdgfrα) were induced in the FB before the appearance of GFP+ cells (Figure 3f), confirming the induction of mesoderm. We detected abundant Fgf10 protein expression in d10 FBs (Figure 3g). We consider the induction of Fgf10 to be important for obtaining limb progenitor identity. This is because during embryonic limb initiation and development, Fgf10 is progressively and restrictively expressed in the prospective limb mesoderm, and Fgf10 initiates and maintains the outgrowth of the embryonic limb bud [30]. By day 14 of induction, most of the GFP+ cells were also expressing α-SMA+ (Figure 3h), but not beta III tubulin (data not shown), confirming they were mesenchymal cells. In the d14 FB cultures, limb field-related proteins, such as isl1, Gli3, Tbx5, Pitx1 and Tbx4 were all detectable (Figure 3i). Notably, although Tbx5 was expressed in most of the GFP+ cells, Pitx1 and Tbx4 proteins were only detectable in a small portion. Thus, we concluded that our induction protocol generated limb progenitor cells of both fore- and hind-limb identity, but with a bias toward forelimb.

**Figure 4:**
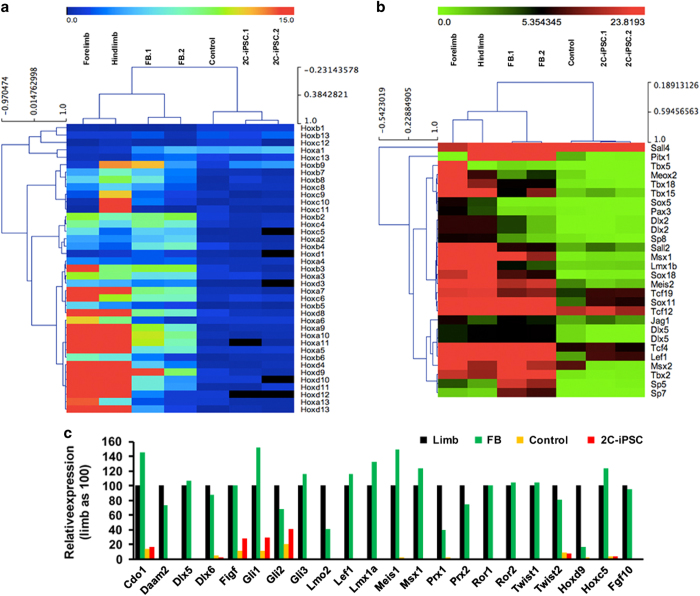
Gene expression analysis of induced limb progenitor cells in comparison with embryonic limb bud. (**a**, **b**) Hierarchical clustering of Hox genes (**a**) and transcription factors related to embryonic limb development (**b**) in FBs, E10.5 fore- and hind-limb, control FB without differentiation (kept in MES-Lif), and 2c-iPSCs. (**c**) Expression levels of selected genes involved in the determination and development of embryonic limb, showing that these genes were induced in differentiated FBs, but not in control or iPSCs.

To further compare our induced FBs to embryonic limb buds, we performed transcriptome analysis and examined the expression levels of genes that have been implicated in limb development [31]. These limb development-related genes are listed in Supplementary Tables S2 and S3. Most of the genes known to be involved in limb development were induced in the FBs, with many genes upregulated over twofold (colored in green, Supplementary Tables S2 and S3). Hierarchical clustering of *Hox* genes, which are involved in limb specification and patterning, showed that our induced FB buds were closely related to E10.5 embryonic limb cells (Figure 4a). We compared a subset of other transcription factors that have important roles in embryonic limb development and patterning, such as *Dlx*, *Lmx* and *Sox* gene family members, by clustering. This also showed that the induced FBs cluster with E10.5 embryonic limbs (Figure 4b). In addition, the levels of gene expression in the induced FBs were comparable to the E10.5 embryonic limbs (Figure 4c). *Fgf10* was also induced to a level equivalent to the embryonic limbs. Thus, these results argue that the green cells we derived from the FBs are closely related to limb progenitor cells. We call them limb progenitor-like cells.

### Induced limb progenitor-like cells promote adult P2 regeneration

**Figure 5:**
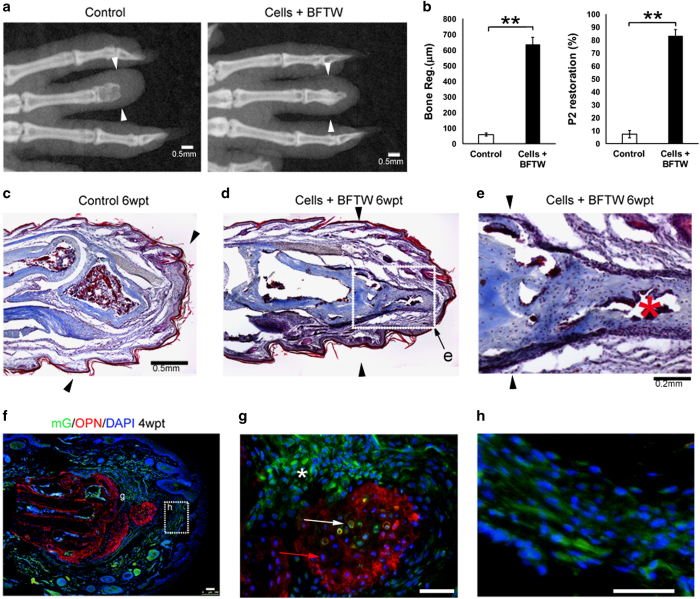
Stimulated adult mouse D3P2 regeneration after transplantation of iPSC-derived limb progenitor-like cells. (**a**) Examples of adult mouse D3P2 with or without limb progenitor-like cell +BFTW transplantation), by X-ray imaging. (**b**) Statistical analysis of length of bone regeneration and P2 restoration in adult D3P2 with graft (limb progenitor-like cells+BFTW) or without (control). Error bars: standard error, *N*=9. **P<0.01, Student’s *t*-test. (**c**–**e**) Sections of D3P2 with Trichrome staining, in control (**d**) or cells+BFTW group (**d**, **e**), 6 wpt. Black arrows indicate amputation levels. White dotted boxes indicate areas shown in (**e**). (**e**) An enlarged view of the P2 regenerate, showing well-integrated outgrowth of the P2 bone, and marrow formed in the regenerated bone (red asterisk). (**f**, **g**) Contribution of GFP+ iPSC-derived limb progenitor cells (exemplified by the white arrow) and GFP− host cells (exemplified by the red arrows) in the bone regenerate (marked by OPN expression, in red). *Indicates areas of GFP+ cells outside the bone. (**h**) Contribution of GFP+ iPSC-derived limb progenitor cells in the connective tissues of the adult D3P2. Scale bars: 0.5 mm in (**a**, **c**, **d**), 0.2 mm in (**e**), 50 μm in (**g,** **h**).

Based on the above observations, we reasoned that the green cells in 2-week-old FBs were suitable for transplantation into the adult mouse P2. However, we found that procedures involving dissociating and sorting the green cells from FBs caused too much damage, resulting in rapid cell loss after transplantation. So, we isolated GFP+ cell clumps from the FBs by removing as many as possible non-green areas under the fluorescence dissecting microscope. Transplantation of the iPSC-derived limb progenitor-like cell clumps together with BFTW significantly enhanced adult P2 regeneration, as shown by both X-ray imaging and histology of the P2 stumps (Figure 5a–e). Measurement of the regenerates confirmed the effectiveness of these cells in promoting regeneration, with an average restoration of >80% of the original D3P2 bone length (Figure 5b, *n*=9). In the regenerated P2, the regenerated bone integrated nicely to the bone stumps, and there was marrow formed within it (Figure 5e, red asterisk). This suggested that endochondral ossification is involved in the new bone formation. Consistent with this, we observed hypertrophic chondrocytes in specimens transplanted with embryonic limb progenitor cells (Supplementary Figure S3).

Although the dimmer expression of membrane-bound GFP compared with nuclear GFP made it harder to calculate the exact contribution of transplanted cells, we did observe GFP+ cells in the bone regenerates (Figure 5f). As in our transplantation experiment using embryonic limb cells, the stimulated bone regenerate contains both GFP+ donor cells (white arrow, Figure 5g) and GFP− host cells (red arrow, Figure 5g). GFP+ cells were also found in the soft tissues (area surrounding the asterisk in Figure 5g and h). Thus, our results showed that the limb progenitor-like cells derived from *Prx1Cre:mT/mG* iPSCs resemble embryonic limb bud cells in stimulating regeneration of the middle phalanges in the adult mouse.

## Discussion

Here we established a protocol for deriving limb progenitor-like cells from iPS cells, and used the adult mouse middle phalange as a model to explore methods for stimulating regeneration. We did not use neonatal mice because neonatal mice are still growing their limbs and have greater regenerative capacity in various organs, such as the neonatal heart, than the adults [32]. There is also significant hypertrophy of the bone stump after amputation in the neonatal mice, thus rendering them unsuitable for determining methods for regeneration stimulation [19]. By contrast, the P2 length in mice of 8 weeks of age and older does not increase (Supplementary Table S1). Thus, we consider that the non-regenerating adult P2 is a better model for studying methods for stimulating regeneration.

The key procedure for generating limb progenitor-like cells in our protocol is the culturing of iPS cells in FBs. In our hands, monolayer culture, or culturing of iPS cells as embryoid bodies, barely produce any GFP+ cells. This suggests that the FB provides a suitable three-dimensional environment for the induction of limb progenitor cells. During the optimization of this protocol, we also found that Bmp4 and retinoic acid treatment, often used to induce mesoderm from iPSCs [25], were dispensable for GFP induction in our FBs. And, more importantly, we found that SB431542 treatment significantly increased the percentage of GFP+ cells in the FBs. The timing of SB431542 and Purmorphamine treatment was also critical, as their presence before day 6 inhibited GFP activation. Therefore, our current protocol represents the minimal treatment for the iPS cells required to generate limb progenitor-like cells. We found CK14 and Fgf8 are expressed in the outer layer of the bud-like structure in the FB cultures (data not shown), suggesting that an active ectoderm is also induced by our differentiation method. Based both on morphology and transcriptome analysis, the limb progenitor-like cells are comparable to embryonic limb mesenchyme cells (Figures 3 and 4), and, like embryonic limb mesenchyme cells, could promote regeneration of the adult mouse P2.

The ability to regenerate the distal digit tips in the mouse provides us with an opportunity for exploring mechanisms to stimulate regeneration in the more proximal levels. Lineage tracing analysis has shown that mouse digit tip regeneration is carried out by multiple types of progenitor cells [9, 11]. Therefore, to promote regeneration, a plausible approach is to provide the stump with all of these multiple types. One way to do this is transplanting cells that can generate the required progenitor cells after transplantation. Our previous work showed that cells with the identity of limb progenitors are the best choice for this purpose [18]. So in the current study, we also used limb progenitor cells for transplantation. The transplanted limb progenitor-like cells contributed not only to the bone, but also to the connective tissues. Besides the GFP+ cluster of cells found underneath the skin (Figure 2), we also observed GFP+ cells in the proximity to a group of cells in continuity with the severed ligament and tendon (Supplementary Figure S4). Although we could not find a good antibody to identify these cells, these observations indicate that the transplanted limb progenitor cells are able to generate several cell types.

To further improve the adult P2 regeneration, we are also considering adding other types of cells to better mimic the regeneration-competent cell compositions found in the regeneration of the terminal phalange (P3) tips. Recent work showed that like amphibian limb regeneration, mouse digit tip regeneration is also regulated by innervation [33]. Nerve-associated Schwann cell precursors regulate digit tip regeneration by secreting factors that promote mesenchymal cell expansion [34]. The importance of the nail organ in digit tip regeneration has also been known for decades [12, 35]. Thus, additional cell types, such as neural progenitor cells and nail stem cells, may further improve the proximal phalange regeneration in the adult mouse.

Ultimately, we expect that the transplanted cells can also stimulate host cells, so that the newly regenerated tissue and organ can seamlessly integrate with the host. In our experiments, this has been observed at least in the bone, where host cells contribute substantially after progenitor cell transplantation (cells+BFTW groups, Figures 2 and 5).

It would be ideal if regeneration could be stimulated by simpler methods, such as the application of additional growth factors to the stump. Unfortunately, at least in our hands, addition of growth factors to the adult mouse P2 stumps is not sufficient by itself to induce complex regeneration. But clearly these exogenous factors are important, as they are required for the survival and proliferation of the transplanted progenitor cells (Figures 2 and 5). In this study, we identified that BFTW is the optimal combination of growth factors for the limb progenitor cells. Wnt3a and Fgf8 increase proliferation of the embryonic limb cells, both *in vitro* and *in vivo* after transplantation (Supplementary Figure S1, Figure 1). This is consistent with their roles in promoting proliferation of embryonic limb progenitor cells and maintaining the cells in an undifferentiated state [36, 37]. Application of Bmp and thymosin beta 4 have been shown to be important for bone formation [18]. The combination of all these factors, BFTW, could efficiently support the transplanted limb progenitor cells, so that the adult P2 can be stimulated to regenerate (Figures 2 and 5). Additional factors, such as neurotrophic or paracrine factors, may further improve the regeneration in the adult mouse phalange and limb.

## Materials and Methods

### Animal husbandry

All animal procedures were conducted in accordance with the guidelines of National Institutes of Health and were approved by the Institutional Animal Care and Use Committee at the University of Minnesota. The D3P2 of athymic nude mice (age 8–10 weeks) was amputated at the middle level under anesthesia. The stump was covered with a piece of Hyphecan membrane (www.hyphecan.com) or Vetbond (3 M) to facilitate healing. The D3P2 stumps were re-opened with a microsurgical knife, 10–12 days after amputation (after the scab fell off) for transplantation. For controls, the D3P2 stumps were re-opened, but were not supplied with transplant. Mice were observed 3 days later under a fluorescence microscope to confirm the success of the transplantation procedure. Mice that failed to retain the fibrin implant were excluded from further analysis. Regeneration of the mouse D3P2 was followed by X-ray imaging using an *Ex Vivo* Extreme small animal imaging system (Bruker, Billerica, MA, USA), for up to 20 weeks post transplantation.

### Embryonic limb progenitor cell culture and analysis

Embryonic limb progenitor cells were isolated from E10.5 mouse embryo limb buds. After manual removal of the epidermis, the mesenchyme of embryonic limb buds was dissociated with TrypLE solution (Life Technologies, Carlsbad, CA, USA) by incubation for 20 min at 37 °C. The dissociated limb bud cells were washed with phosphate-buffered saline (PBS) and re-suspended in Dulbecco’s modified Eagle’s medium containing growth factors or small molecules. One (as in Supplementary Figure S1a), or a combination (as in Supplementary Figure S1b) of the following growth factors (R&D Systems, Minneapolis, MN, USA, ng/ml) or small molecules (Sigma-Aldrich, St Louis, MO, USA) were tested: Activin A (10), Bmp2 (10), Bmp4 (10), Bmp7 (10), Bmp9 (10), Fgf2 (5), Fgf8 (5), Fgf10 (10), Shh (5), Wnt3a (10), Wnt5a (50), thymosin beta 4 (5), CHIR99021 (1 μM), BIO (50 nM) and Purmorphamine (4 μM). Cell proliferation assays were performed on day 3 cultures with Cell Proliferation Reagent WST-1 (Roche, Penzberg, Germany) as instructed. For migration analysis, cells were mixed in fibrin gel and plated onto plastic. The distance of cell migration out of the fibrin gel was measured after 5 days culture, in the presence of various growth factors (as above).

### Fibrin matrix/cell transplantation

Embryonic limb progenitor cells were collected from E10.5 *nucGFP* transgenic embryos as above. After brief centrifugation and wash with PBS, the limb progenitor cells were re-suspended in fibrinogen solution (40 mg/ml; F8630, Sigma-Aldrich). The fibrin solution was then mixed 1:1 (volume) with Thrombin (80 units/ml, T7009, Sigma, supplemented with CaCl_2_) by brief pipetting. The mixture usually solidified within 30 s to form a fibrin gel. The fibrin gel was flooded with warm Dulbecco’s modified Eagle’s medium and trimmed, inserted underneath the newly re-opened stump wound epithelium. For cells with growth factors, Affi-Gel beads presoaked with BFTW (Bmp2, Fgf8, Thymosin beta 4, Wnt3a, all as 0.1 mg/ml) were implanted to the transplantation site. Beads were incubated in individual factor and implanted separately into the D3P2 stump, one bead at a time, on 4 consecutive days. Approximately 10 thousand cells, in a fibrin gel about 0.4 mm in diameter, were transplanted for each P2 stump.

For transplantation of iPSC-derived cells, GFP+ cell clumps were isolated manually and inserted underneath the wound epithelium. About 15–20 thousand iPSC-derived limb progenitor-like cells were transplanted for each P2 stump.

### 2c miPS cell generation and differentiation of cells to limb progenitor-like cells

*Prx1Cre* and *mT/mG* transgenic mice were crossed to obtain *PrxCre:mT/mG* transgenic mice. Embryonic limb fibroblasts and tail fibroblasts were isolated from the limb and tail of an E14 mouse embryo, following a protocol generating mouse embryonic fibroblasts [38]. Passage 2 cells were used to generate iPSCs, with methods essentially as previously described [20]. iPSC clones were picked, characterized and iPSC lines with a normal karyotype were selected. iPS cells were maintained on irradiated embryonic mouse fibroblasts with mouse embryonic stem cell (mESC) medium (knockout Dulbecco’s modified Eagle’s medium, Life Technologies) supplemented with 10% fetal bovine serum (HyClone, Logan, UT, USA), 10% knockout serum replacer (Life Technologies), 1% l-glutamine 200 mM (Life Technologies), 1% penicillin/streptomycin (Life Technologies), 1% minimum essential medium non-essential amino acids (Life Technologies), 0.1 mM 2-Mercaptoethanol (Sigma-Aldrich) and 1000 Units/ml ESGRO-mLIF (Millipore, Burlington, MA, USA), at 37 °C in 5% CO_2_. The 2c clone of iPS cell lines from the tail fibroblasts was used in this study (2c iPSC).

To initiate limb progenitor cell differentiation, 2c *Prx1Cre:mT/mG* cells cultured in mouse embryonic stem cell medium for 3 days were dissociated to single cells with 0.05% Trypsin-EDTA (Gibco), and re-suspended in complete mouse embryonic stem cell medium. Feeder cells in the iPS cell cultures were depleted by re-plating the cells on tissue culture plates, and incubation for 45 min at 37 °C and 5% CO_2_. After counting, the cells were washed with PBS and re-suspended in fibrinogen solution (0.2 million per microliter). Hemispherical FBs were then made by mixing 1.5 μl of cells with 1.5 μl of Thrombin (80 units/ml, T7009, Sigma-Aldrich) by pipetting up and down on the cover of tissue culture plates. FBs were then transferred manually to an Ultra-Low Attachment plate (Costar, Corning, NY, USA) in mouse embryonic stem cell medium without leukemia inhibitory factor for 3 days. On day 3, the basal medium was switched to mesenchymal stem cell medium (Life Technologies), with the addition of 10 ng/ml of Fgf8 and 3 μM of CHIR99021 (Sigma-Aldrich). From day 7 onward, the medium was supplemented with Fgf8 (R&D Systems, 10 ng/ml), CHIR99021 (3 μM), Purmorphamine (Sigma-Aldrich, USA, 4 μM) and SB431542 (Sigma-Aldrich, USA, 2 μM), and changed every 3 or 4 days.

### RT-PCR, immunofluorescence, flow cytometry, EdU assay and cell counting

Total RNA was extracted with Trizol reagent (Life Technologies) and treated with Turbo DNase I (Ambion, Austin, TX, USA) before being reverse-transcribed into complementary DNA with the Superscript III RT system (Life Technologies). Real-time PCR was performed on an Eppendorf Light-cycler with SYBR green real-time mix (Roche-Aldrich, St Louis, MO, USA). Expression of genes of interest was normalized to glyceraldehyde 3-phosphate dehydrogenase, and relative expression levels were presented in Figure 4. Primers used are listed in Supplementary Table S4.

For immunohistochemistry analysis, phalanges were fixed in Leica 4% paraformaldehyde (PFA) (Sigma), decalcified in 0.4 M EDTA in PBS for several weeks, embedded in optimum cutting temperature (OCT) compound and sectioned. FBs were fixed in 4% PFA and sections were prepared. Sections were permeabilized with 1% Triton X-100 in PBS for 15 min, and blocked with blocking buffer (PBS containing 1% BSA and 0.3% Triton X-100). After incubation of primary antibody (usually diluted 1:200), sections were washed and incubated with secondary antibodies (1:500 dilution), washed again counterstained with 4,6-diamidino-2-phenylindole, and mounted.

Primary antibodies used were Kdr (R&D Systems, AF644), PDGFRa (R&D Systems, AF1062), Fgf10 (R&D Systems, AF6224), Isl1 (Abcam, Cambridge, MA, USA, 109517), Gli3 (Abcam, ab6050), α-SMA (Sigma-Aldrich, A2547), Tbx5 (Abcam, 137833), Pitx1 (Sigma-Aldrich, HPA008743), Tbx4 (Novus, St Louis, MO, USA, nbp2–58195), OPN (R&D Systems, AF1433), Col2a1 (R&D Systems, AF3615). Secondary antibodies were Alexa Fluor 555, Alexa Fluor 647-conjugated IgGs (Invitrogen, Carlsbad, CA, USA).

For flow cytometry analysis, the FBs were dissociated with TrypLE to single cells, and analyzed with a Fortessa X-20 analyzer, using 488B (for GFP) and 561C (for tdTomato) filters.

Cell proliferation analysis were performed on sections of D3P2 with Click-it EdU assay kit (Invitrogen) as instructed. EdU (50 mg/kg) was administered to the animals intraperitoneally 48 h before specimen collection. GFP+ and EdU+ cells were counted on at least three non-adjacent sections of three separate specimens.

### Transcriptome analysis

Three batches of GFP+ and tdTomato+ cell clumps from D14 FBs, 2c-iPSCs or non-induced FBs, were dissected in 0.05% Trypsin-EDTA, and pooled for total RNA extraction with TRIZol Reagent (Invitrogen). Sample collection was performed twice to generate biological replicates. RNA sequencing was performed by Biozeron Ltd., Shanghai, China. Libraries were constructed with Illumina Truseq RNA sample prep kit (Illumina, San Diego, CA, USA), amplified for 15 cycles, purified with low range ultra agarose and sequenced on Illumina Hiseq platform. Data were processed and analyzed with SeqPrep, TopHat, Cuffdiff, and viewed with IGV. RPKM (Reads Per Kilobase of transcript per Million) (FPKM, Fragments Per Kilobase of transcript per Million ) values were obtained with cufflinks. Hierarchical cluster analysis was performed after Person correlation efficiency, and images presented in Figure 4 were generated with MeV software (Rockville, MD, USA) (mev.tm4.org).

### Image acquisition and processing

Regeneration of the mouse D3P2 was observed under a Leica MZ16F microscope equipped with a Qimaging Retiga 2000R digital camera (Qimaging, Surrey, BC, Canada). X-ray images were captured using the *Ex Vivo* Extreme small animal imaging station (Bruker). Micrographs of immune stained sections were captured with DMI4000 or DMI6000B microscopes equipped with digital cameras (Leica, Wetzlar, Germany). The microscopes were equipped with A4 (for 4,6-diamidino-2-phenylindole), L5 (for GFP), N2 (for tdTomato, Alexa Fluor 555) and Y5 (for Alexa Fluor 647) filter cubes. Figures were prepared with Photoshop (Adobe, San Jose, CA, USA).

### Regeneration measurements

To analyze bone and soft tissue regeneration, X-ray images were measured with the ruler tool of Photoshop software (Adobe). The lengths of D2P1 (Digit 2, Phalange 1), D2P2, D4P1, D4P2, D3P1, D3P2 and D3P2R bones were measured for all animals. Ratios of P2/P1 in D2–4 were calculated and used to determine the expected length of D3P2: D3P2 (expected)=D3P1×P2/P1 ratio. Overall P2 restoration was then determined as (D3P2 (measured)+soft tissue (measured))/D3P2 (expected)×100%.

### Statistics

Bone and soft tissue regeneration measured from X-ray images were analyzed between groups by student *t*-test, two-tailed, unpaired. Regeneration of multiple groups was analyzed with analysis of variance (one way). *P*<0.01 is statistically significant.
