# Supplementary material for: Generation of iPSC-derived limb progenitor-like cells for stimulating phalange regeneration in the adult mouse
Source: Cell Discov. 2017 Dec 19;3:17046–. doi: 10.1038/celldisc.2017.46 (PMC5735367; doi:10.1038/celldisc.2017.46)
Supplement: Supplementary Information [file celldisc201746-s1.pdf]

Supplementary Information

Generation of iPSC-derived limb progenitor-like cells for stimulating phalange regeneration in the adult mouse

Ying Chen[a,1,2], Hanqian Xu[b,1], and Gufa Lin[a,b,2]

[a]Stem Cell Institute, Department of Genetics Cell Biology and Development, University of Minnesota, Minneapolis, MN 55455, USA; and

[b]Research Centre for Translational Medicine, Shanghai East Hospital, School of Life Sciences and Technology, Tongji University, Shanghai 200120, China.

INVENTORY OF SUPPLEMENTARY INFORMATION

Supplementary Figures

Supplementary Figure 1. Identification of factors for transplanting embryonic limb progenitor cells.

Supplementary Figure 2. Generation of *Prx1Cre:mT/mG* iPS cells.

Supplementary Figure 3. Hypertrophic chondrocytes in P2 stump.

Supplementary Figure 4. GFP cells contribute to connective tissues in adult D3P2 regenerate.

Supplementary Tables.

Supplementary Table 1. Length of Phalange 1 (P1) and Phalange 2 (P2) and ratio of P2/P1 in Digits 2-4.

Supplementary Table 2. Expression values of genes known to function during limb development, or family members of signal transduction pathways known to contribute to limb patterning.

Supplementary Table 3. Expression values of 279 genes identified by Taher et al.

Supplementary Table 4. PCR primers used in this work.

Supplementary Figures

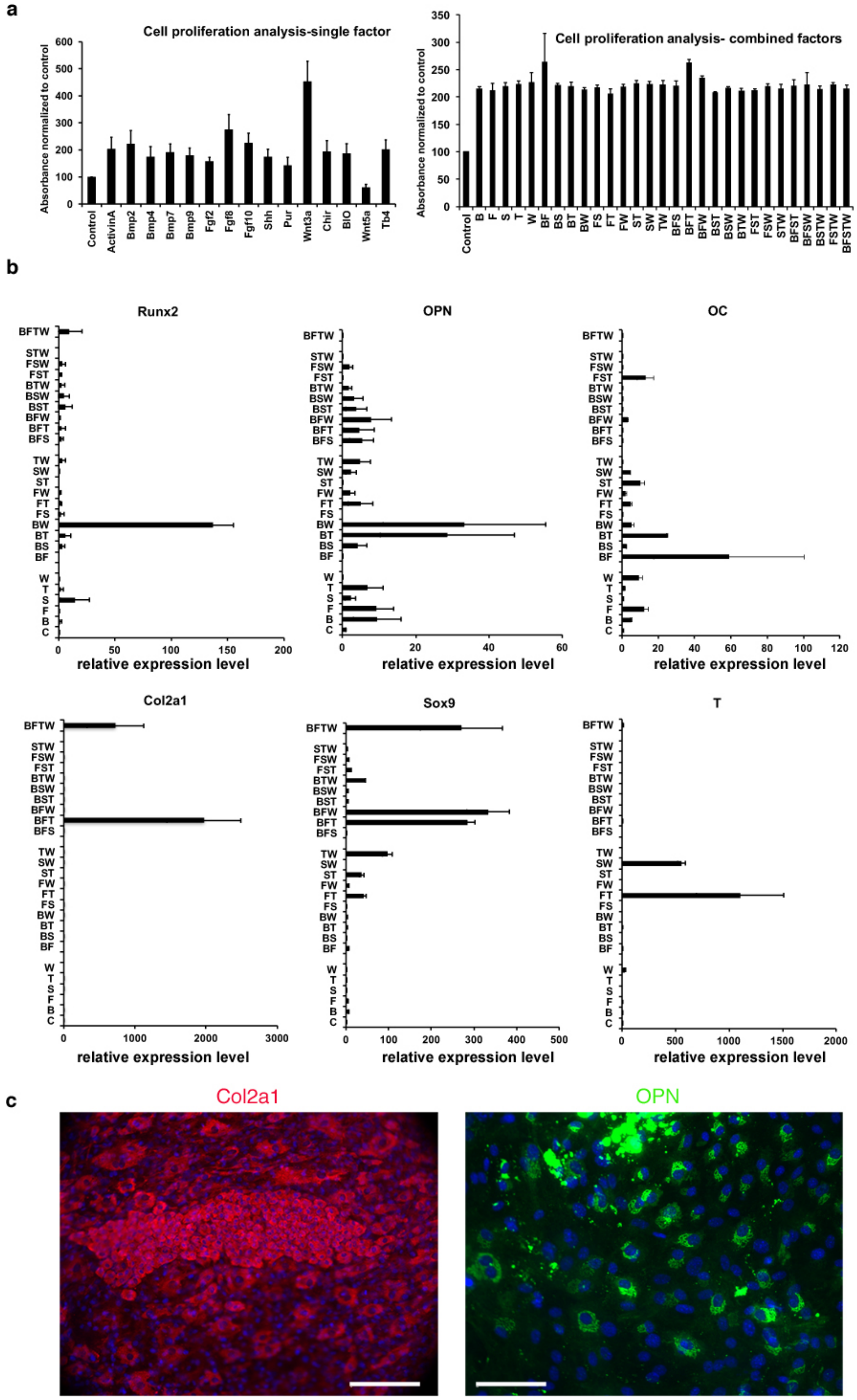

Supplementary Figure 1 Identification of factors for transplanting embryonic limb progenitor cells. (**a**) Analysis of proliferation of E10.5 embryonic limb cells treated with single growth factors or small molecule, or combination of growth factors. Cell proliferation was analyzed with WST-1 reagent and normalized to control. Abbreviations: B = Bmp2, F = Fgf8, W = Wnt3a, T = Thymosin beta 4, S = Shh. (**b**) Detection of osteogenic markers *Runx2*, *OPN*, *Osteocalcin* (*OC*) and chondrogenic lineage markers *Collagen 2a1* (*Col2a1*), *Sox9*, and mesodermal marker *T* in E11 cells treated with growth factors. (**c**) Immunofluorescence detection of Col2a1 and OPN in cells treated with growth factors. Shown are the example of cells treated with BFTW (Bmp2, Fgf8, thymosin beta4 and Wnt3a). Scale bars: 100 μm.

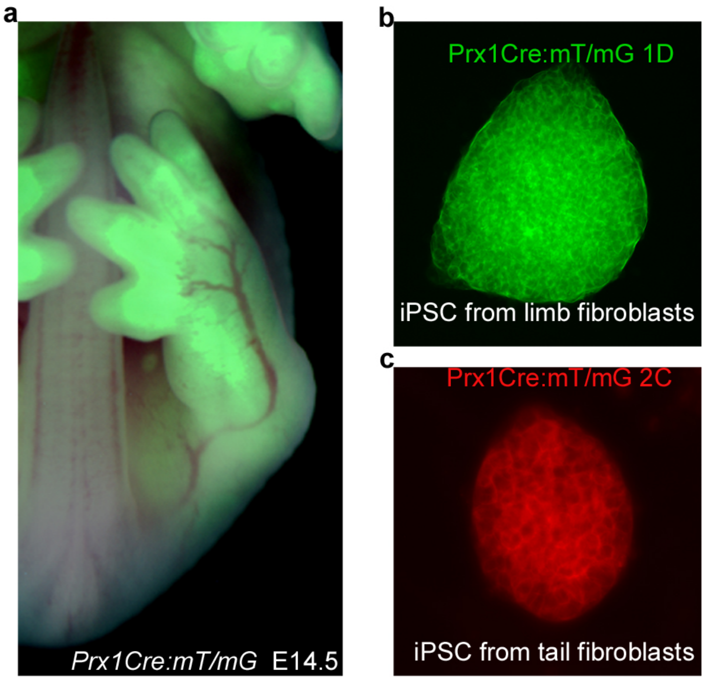

Supplementary Figure 2 Generation of *Prx1Cre:mT/mG* iPS cells. (**a-c**) *Prx1Cre:mT/mG* transgenic mouse, crossed from *Prx1-Cre* and *CMV-mT/mG* mice, at E14.5 (**a**), was used to generate iPS cells from the limb mesenchymal cells (**b**, expressing membrane GFP) and tail fibroblasts (**c**, expressing membrane tdTomato).

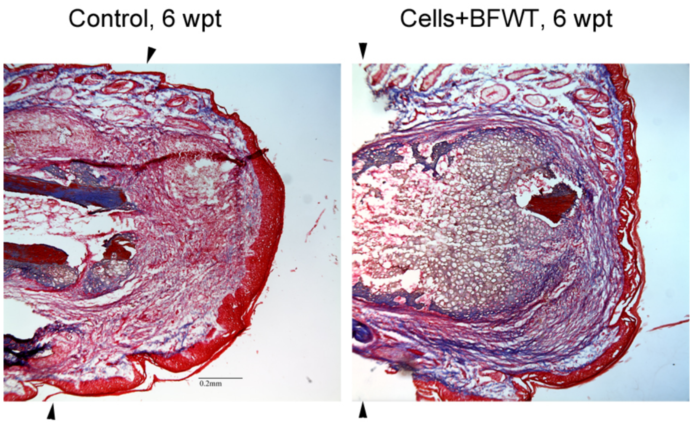

Supplementary Figure 3 Hypertrophic chondrocytes in P2 stump after transplantation of embryonic limb progenitor cells together with BFTW, but not in controls. Black arrowheads indicate amputation levels in control. Scale bar 0.2 mm.

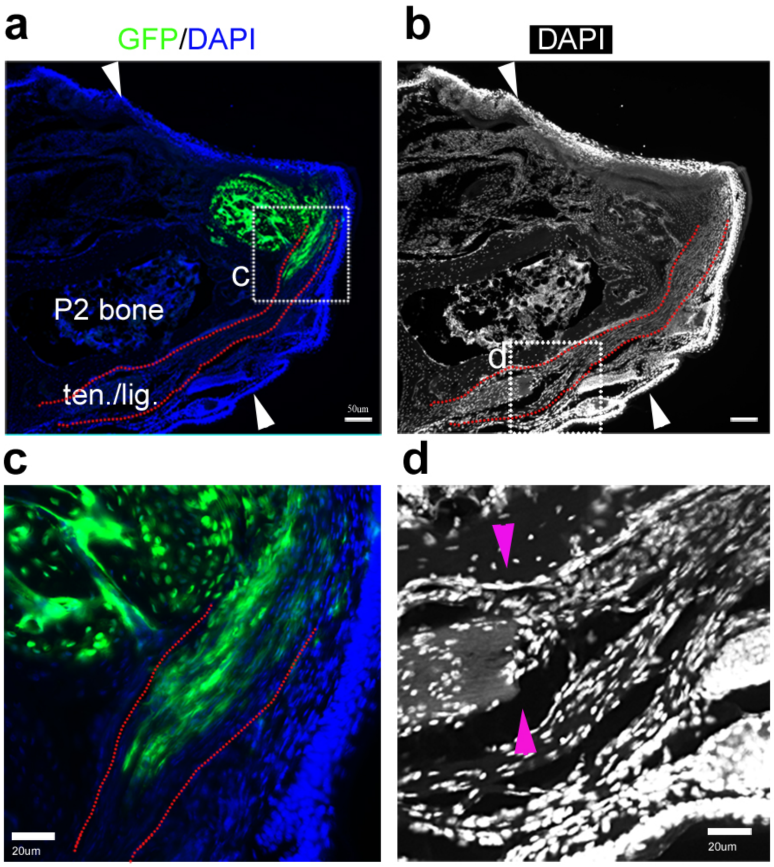

Supplementary Figure 4 GFP cells contribute to connective tissues in adult D3P2 regenerate. (**a**,**b**) A parasagittal section of a D3P2 regenerate, with GFP+ cells shown in green, nuclei shown in blue (**a**) and in white (**b**). The red dotted line outlines a population of cells in continuity with the severed ligament and tendon. (**c**) Enlarged view of the GFP+ cells located in the distal connective tissue. (**d**) Enlarged view of the area where the ligament and tendon were amputated. Scale bar: 50 µm in (**a**,**b**), 20 µm in (**c**,**d**).

Supplementary Tables.

Supplementary Table 1.

Length of Phalange 1 (P1) and Phalange 2 (P2) and ratio of P2/P1 in Digits 2-4

Age (Weeks)	Average length of phalange (μm)						Average P2/P1 (%)		
	D2P1	D3P1	D4P1	D2P2	D3P2	D4P2	D2	D3	D4
20	2893	2940	2900	1604	1646	1624	55.46	55.99	56.00
22	2942	2944	2935	1603	1649	1629	54.49	56.00	55.50
24	2897	2863	2911	1618	1547	1663	55.85	53.99	57.13
26	2850	2896	2853	1585	1636	1573	55.63	56.49	55.13
28	2905	2925	2912	1610	1594	1598	55.40	54.49	54.87
34	2890	2954	2898	1611	1698	1607	55.74	57.49	55.45
36	2874	2928	2880	1561	1625	1587	54.30	55.50	55.10
40	2961	3010	2949	1639	1701	1640	55.34	56.49	55.59
49	3035	3110	3045	1640	1726	1680	54.04	55.49	55.18

Note: Measurements taken from 6 untreated adult mice.

Supplementary Table 2. Expression values of genes known to function during limb development, or family members of signal transduction pathways known to contribute to limb patterning (Taher L, Collette NM, Murugesh D, Maxwell E, Ovcharenko I, Loots GG. Global Gene Expression Analysis of Murine Limb Development. Emanueli C, editor. PLoS ONE. 2011 Dec 9;6(12):e28358–60**).** Genes with FPKM <5 in all of the samples were not considered. Genes that are differentially expressed between control and induced fibrin bodies are highlighted in green (fold change >=2 or <=0.25). Compared to un-induced control fibrin bodies, upregulated genes in induced fibrin bodies are shown in green. Of these, genes that have the similar expression to the limb are highlighted in bode green (fold change between 0.25 to 2).

Gene	Forelimb	Hindlimb	FB.1	FB.2	CTL	2C-iPSC.1	2C-iPSC.2
			BMP signalling				
Bmp1	41.1163	30.4401	15.778	15.6434	20.5796	9.06214	6.42435
Bmp2	2.1158	1.0559	9.5837	6.92013	2.96549	0.306039	0.226085
**Bmp4**	**42.2176**	**36.7386**	**65.0721**	**52.2874**	**8.1654**	**3.90273**	**4.06574**
Bmp5	1.81542	1.46024	8.69698	8.95021	0	0.0275568	0
Bmp6	0.613827	0.542468	7.74299	7.51293	2.66561	1.86078	1.78721
**Bmp7**	**22.8271**	**21.7391**	**13.5691**	**14.0194**	**4.6323**	**5.93613**	**6.02421**
Bmpr1a	48.2772	43.5512	40.3985	38.3971	31.2364	22.8855	21.8785
Bmpr1b	3.51768	2.2461	0.30357	0.416357	0.0127711	0.0299887	0.0565046
**Bambi**	**9.28003**	**10.0213**	**9.56697**	**6.67895**	**1.73753**	**5.39703**	**4.90676**
Grem1	18.898	20.0018	11.8786	10.7486	5.75164	0.742682	0.619742
Grem2	0.14793	0.0119994	2.85316	4.34277	3.26785	1.73355	0.991053
Bmper	0.358448	0.0957309	1.7852	1.70001	2.52792	0.315349	0.300124
Bmpr2	12.1338	10.8143	14.4404	11.2239	10.0284	5.21065	5.54421
Gdf2	0	0	0.0713398	0.0343359	0	0	0
Gdf3	0	0.050991	5.53476	9.70384	19.0493	22.9353	21.301
Gdf5	0.913211	0.236897	0.116368	0.0897735	0.151789	0.260373	0.284933
Gdf10	0.84128	0.268137	51.3159	47.3619	0.176177	0.0390354	0.0397767
Nog	0.438899	0.488936	6.6218	5.09084	8.74624	0.628629	0.501818
			TGF-beta signalling				
Tgfb1	6.89925	7.44798	7.43439	7.82643	4.31869	4.33263	3.44472
Tgfb1i1	5.6971	4.43484	16.1018	19.7766	9.98826	1.96006	1.91155
**Tgfb2**	**8.8936**	**2.45205**	**10.0738**	**12.0774**	**0.805915**	**0.366839**	**0.465031**
Tgfb3	7.01735	5.3703	2.30691	2.99435	0.893279	1.57054	1.2826
Tgfbi	15.2798	9.77302	47.1929	59.9915	3.58767	1.69261	1.19499
Tgfbr1	60.957	64.2437	16.4238	15.8806	12.0594	11.8847	9.58057
Tgfbr2	6.61189	6.8938	9.78798	9.33589	12.1532	2.43059	1.46519
Tgfbr3	4.80494	4.24422	19.6985	14.4288	18.6098	3.74457	2.62582
			Hedgehog signalling				
**Gli1**	**18.9221**	**16.6412**	**28.3918**	**25.6462**	**1.97277**	**6.16965**	**4.18739**
**Gli2**	**17.1178**	**18.7088**	**13.2044**	**11.2478**	**3.55538**	**8.35933**	**6.22358**
Gli3	45.8305	53.3122	8.58039	6.94927	1.14943	2.83141	1.43103
Shh	9.40159	7.20801	1.14964	0.921135	0.0195031	0.12	0.0202665
Ihh	0.0466544	0.167422	30.8763	25.0125	6.38075	0.150855	0.384396

**Ptch2**	**6.81732**	**4.90773**	**4.39102**	**5.37708**	**2.19952**	**5.83271**	**6.34022**
Ptch1	21.6036	20.4879	51.809	44.6577	14.3588	35.1278	30.6699
				Fgf signalling			
Fgf2	1.48826	0.757688	0.891656	0.702374	0.326392	0.656024	0.152977
Fgf3	0.266177	0.214751	1.12616	0.951847	4.38775	0.999027	2.57397
Fgf4	1.86172	1.57053	8.40645	12.9527	51.933	106.927	114.041
Fgf5	0.382965	0.350881	0.310712	0.108404	0.543958	0.476246	0.510554
Fgf6	0	0	0.0463519	0.044384	0	0.0109628	0
Fgf7	0.0775283	0.016899	0.256194	0.131633	0.798674	1.00663	0.644433
Fgf8	69.6968	75.7258	1.56978	0.303374	0.405132	1.91589	0.602125
Fgf9	1.78063	0.596221	0.161094	0.224081	0.116906	0	0.0336944
Fgf10	12.902	12.2524	3.31859	3.03151	3.55465	0.846225	0.579062
Fgf11	0.4543	0.144819	12.4321	9.83742	2.01678	6.28716	4.8598
Fgf12	0.0960669	0	0.12404	0.267456	0.0347701	0	0
Fgf13	3.70648	2.49971	1.52548	1.05713	0.211856	1.47795	0.917202
Fgf15	0.187638	0.221193	1.96449	4.66697	2.85801	3.16458	2.78811
Fgf17	0.418086	0.745639	0.63193	0.617159	0.925568	4.26915	3.02963
Fgf18	0.971816	0.419921	0.142451	0.0687277	0.125288	0.639095	0.514802
Fgfbp1	0.380412	0.0378878	6.77898	11.9033	11.8247	8.11797	6.9789
Fgfbp3	2.22071	2.08695	4.87074	3.9873	1.26585	1.68267	2.80824
**Fgfr1**	**92.6589**	**80.3167**	**127.984**	**106.587**	**31.8183**	**49.5417**	**42.3615**
Fgfr1op	22.9171	23.415	17.6359	19.2189	18.4851	31.1748	33.9446
Fgfr1op2	34.9704	34.5044	34.6165	35.3808	30.3356	35.687	35.666
Fgfr2	19.7252	15.0701	44.6145	34.1822	8.41835	4.13334	3.86136
Fgfr3	2.85275	1.36994	6.81378	5.65942	10.2451	1.21247	1.80415
Fgfr4	0.634208	0.122929	6.3285	4.85212	4.25137	3.5335	4.00052
				ROR signalling			
**Ror1**	**6.40526**	**6.47268**	**4.17708**	**3.40544**	**0.557197**	**0.0882142**	**0.0682878**
**Ror2**	**37.2502**	**38.9415**	**24.5556**	**25.0132**	**2.05418**	**3.84918**	**3.35404**
				Wnt signalling			
Wnt1	0	0	0.0504951	0.0489436	0.251133	0.0229993	0
Wnt2	0.35319	3.97549	0.561628	0.327051	0.0229917	0.115813	0.0229217
Wnt2b	0.486403	0.774248	0.383241	0.304795	0.620087	0.29605	0.253378
Wnt3	3.27994	2.80716	0.297951	0.354052	0.149052	0.310951	0.34574
Wnt3a	0.0621071	0	0.0422144	0	0.0587868	0.0991995	0.244115
Wnt4	2.74924	2.66876	5.69687	4.18242	1.29194	0.564534	0.639001
Wnt5a	55.9313	40.1678	14.6161	13.3926	1.67552	0.795035	0.55928
Wnt5b	11.9207	11.2737	2.24403	2.35839	0.32861	0.392469	0.0643258
Wnt6	26.0981	20.5897	3.02657	2.30985	0.681776	0.532431	0.594223
Wnt7a	4.90943	4.78793	0.313289	0.265261	0.840938	0.121928	0.209446
Wnt7b	7.88962	5.8284	3.65434	3.94504	21.1166	1.58417	3.28589
Wnt8a	0.12933	0.283159	0.430967	0.191449	0.215552	0.183123	0.0310506
Wnt8b	0	0	0.587015	0.435564	0.0313908	0.0156974	0.0157253

Wnt9a	0.39927	0.24166	3.45912	3.34018	3.42868	3.02718	3.39226
Wnt9b	0.799174	0.639473	0.0121365	0.0117007	0.0228576	0.0114228	0
Wnt10a	0.691643	0.549146	1.05209	1.11813	0.108554	0.0896497	0
Wnt10b	2.06602	1.48473	0.159732	0.280619	0.147837	0.105406	0.207136
Wnt11	15.971	6.17571	89.6272	66.4713	2.78214	0.837172	1.72387
Wnt16	1.59311	0.795801	1.44182	0.462924	0.199636	0.0671976	0.226571
Dkk1	5.44566	6.17909	32.9601	27.4774	1.50701	0.39568	0.980884
Dkk2	2.04847	0.995971	3.59318	4.40577	0.172665	0.361789	0.361559
Dkk3	2.14285	1.80275	6.99443	6.81038	6.46641	5.96187	4.09073
Dkk4	0.131098	0.337261	0.0915074	0.0436223	0	0	0.0422822
Lrp1	23.4116	20.3677	25.0608	19.0557	11.0844	44.7146	30.3021
Lrp1b	0.00564225	0	0.0413623	0.0892576	0.0197965	0.0545354	0.0527807
Lrp2	0.0417318	0.0767206	22.5416	23.2702	16.1831	8.00916	7.64074
Lrp2bp	0.0719501	0.0297475	0.207448	0.034063	0.394765	0.315873	0.131339
Lrp3	19.3174	20.3424	5.21036	5.77948	1.44214	3.89153	2.65957
**Lrp4**	**8.38668**	**8.15991**	**6.76346**	**5.8327**	**2.03529**	**2.86026**	**2.00222**
Lrp5	29.5633	24.278	29.101	23.3522	21.4094	28.0696	24.0555
Lrp6	27.6413	24.4911	19.9843	15.5319	18.9993	23.3186	21.9505
Lrp8	6.56273	7.69648	7.70058	8.56668	12.1983	8.98344	7.2487
Lrp10	9.83712	10.8201	26.4388	23.3825	33.8663	15.0525	15.2042
Lrp11	0.936304	1.59321	7.92238	7.8315	12.0708	33.385	35.465
Lrp12	14.0525	13.2626	7.03608	6.38717	3.61275	4.33714	3.93223
Tcf3	239.73	216.125	77.5758	71.8792	52.7012	81.7705	75.6986
**Tcf4**	**60.6172**	**54.6945**	**74.4093**	**60.1254**	**6.90369**	**8.85666**	**6.3081**
Tcf7	28.5281	46.3565	40.2796	35.5226	25.8248	38.6179	46.2082
Tcf7l1	42.3222	37.9325	11.7404	12.6008	40.7595	41.1606	39.555
Tcf7l2	18.2637	15.0159	7.85925	7.00558	18.6704	3.32431	3.84908
Tcfl5	0.853161	1.31312	1.63724	2.5119	11.7819	22.2162	19.473
**Tcf12**	**142.735**	**148.01**	**95.9788**	**85.1101**	**16.671**	**19.9014**	**14.8191**
Tcf15	24.2625	22.0611	3.20663	3.09052	2.99857	11.6885	9.4521
**Tcf19**	**20.0977**	**20.7946**	**11.8378**	**13.5034**	**4.46987**	**8.26853**	**8.59742**
Tcf20	19.1393	19.8577	15.5848	13.3338	21.2335	22.8485	21.136
Tcf21	0	0.641899	0.215192	0	0.0975097	0	0
Tcf23	0	0	1.88295	1.26562	3.82034	0.215251	0.199145
Tcf24	0.0408896	0.056194	0.658345	0.848819	1.15816	1.59565	1.55223
Tcf25	50.929	53.3062	108.003	109.552	76.738	79.1937	77.6308
Fzd1	35.9394	34.2638	11.7598	11.2089	1.89381	2.21363	1.07927
**Fzd10**	**41.3391**	**43.0741**	**52.327**	**30.5844**	**3.86483**	**2.61316**	**2.80629**
Fzd2	109.028	84.4435	25.6997	27.9194	4.50789	9.72899	7.14731
Fzd3	11.4526	12.203	4.77998	2.81201	1.18395	2.83446	2.06036
Fzd4	5.15098	5.11724	1.77453	2.42165	1.14351	1.90044	1.45486
Fzd5	0.803757	0.702388	7.71534	5.92601	8.74904	24.5471	22.0431
**Fzd6**	**4.30522**	**4.65576**	**7.29971**	**4.92151**	**3.02026**	**2.08723**	**1.7898**
Fzd7	36.8867	38.8466	27.3071	26.1454	25.069	49.7789	43.2107
Fzd8	5.02403	5.33793	2.65666	3.26848	1.86372	2.31245	1.28024
Fzd9	0.355179	0.146399	1.04408	1.66699	1.63671	3.1449	2.52988

**Lef1**	**30.8284**	**35.8057**	**37.831**	**31.3818**	**2.28727**	**9.06981**	**8.20697**
Sost	7.94316	7.13728	0	0.0295147	0.111445	0.0854866	0.117364

Transcription factors

Sox1	0.0115368	0	0.202212	0.686705	1.05126	1.88902	1.23684
Sox2	0.498167	0.193591	48.4501	55.6054	113.736	175.229	191.387
Sox3	0	0.164792	1.13026	0.745942	1.32237	6.64476	4.39978
Sox4	113.973	82.1393	31.1478	37.0805	19.5432	14.5644	11.7036
Sox5	6.50545	4.24235	0.37551	0.412874	0.195992	0.0128753	0.0311228
Sox6	2.21362	2.43662	0.40372	1.35327	0.519264	0.0492503	0.116905
Sox7	4.30397	5.44544	4.10008	4.29446	6.67185	0.541524	0.721024
Sox8	5.0172	8.41812	0.171377	0.714158	0.265453	1.43261	1.30099
Sox9	36.9377	20.3915	1.72768	1.60982	1.11611	1.10077	0.827771
Sox10	0.766757	0.1923	0.0609567	0.0395443	0.130167	0.20905	0.370505
**Sox11**	**112.645**	**106.069**	**69.0621**	**53.9496**	**3.40477**	**8.04077**	**6.91498**
Sox12	68.4595	55.4396	17.8175	18.2027	12.3461	24.0953	21.3891
Sox13	16.3977	23.5677	7.78686	6.2643	5.90714	7.69813	7.33587
Sox14	0.690213	0.485243	0.0845927	0.0546279	0	0	0.0917474
Sox15	0.24499	0.142139	9.44542	13.1732	27.6233	28.4674	38.9536
Sox17	6.08338	6.4162	99.6058	86.8578	71.5345	0.994284	2.00075
Sox18	17.7428	24.3378	9.30115	7.04412	0.0336831	0.410631	0.409482
Sox21	0.240907	0.0750004	3.81316	3.4661	4.5028	3.70913	6.94644
Sp1	25.5609	28.6819	32.1227	32.8347	36.8723	59.2289	64.5988
Sp100	0.0457486	0.105291	0.0725747	0	0.476953	0.30509	0.132497
Sp2	13.5848	14.5376	8.39429	9.30781	13.5535	22.6216	21.414
Sp3	46.0433	45.8397	44.0705	43.3881	49.6317	37.8384	45.0381
Sp4	7.97996	6.37753	4.0627	4.1989	3.58967	4.36949	4.15837
Sp5	3.97052	2.17369	19.4949	14.3797	0.17976	0.0799848	0.346333
Sp6	8.24819	10.1207	4.35559	2.55323	1.9775	3.64567	3.98274
Sp7	1.33066	1.0265	11.7925	8.7158	0.0195407	0.0193088	0.0789013
Sp8	6.30727	6.49008	2.55122	1.15645	0.629256	0.295569	0.134238
Sp9	0.304577	0.592259	1.92583	0.979643	0.159434	0.034846	0.0815457
Pax1	13.4157	0.869013	0.157513	0.143549	0.0822346	0	0
Pax2	0.0904443	0.184125	0.155382	0.436931	0.0241924	0	0.0175603
Pax3	5.89473	4.05589	0.751823	0.817375	0.293665	0.0125333	0.0122428
Pax4	0	0	0	0	0	0	0
Pax5	0	0.124073	0.214778	0.16699	0.0433977	0.180602	0.134768
Pax6	0.0373868	0.114682	0.445798	0.640644	0.755433	1.67643	0.901632
Pax7	0.171933	0.135619	0.0179191	0.0258989	0.050608	0.0828146	0.0421799
Pax8	0.0420717	0.0361246	0.457781	0.461315	0.429728	0.285708	0.142661
Pax9	0.440775	0.208223	0.711069	1.05991	0.532443	0.0265685	0.0775256
Sall1	29.4874	23.0166	10.0291	10.0441	17.2738	27.3978	18.9684
Sall2	23.3888	23.7906	7.26305	6.45616	2.01312	3.49259	2.28446
Sall3	13.5222	9.46549	0.99487	0.631397	0.0786346	0.555944	0.540468
Sall4	17.4844	27.1282	25.75	23.5963	50.1525	75.8042	78.1869
Msx1	130.815	162.33	30.272	14.2444	0.416495	0.854331	0.774057

**Msx2**	**23.8193**	**14.6941**	**29.9828**	**16.4369**	**10.0444**	**0.545068**	**0.465049**
Msx3	0.0803319	0.0475325	0.82367	1.94111	0.355078	0	0.0789988
Meis1	22.5852	33.7943	8.55588	9.95732	5.99392	0.963567	0.9801
Meis2	37.5164	57.6914	16.254	19.0293	2.97226	1.11425	1.55158
Meis3	4.50534	10.6087	8.71088	7.41972	5.44191	1.25528	1.3392
Meox1	4.47254	6.36929	0.0467142	0.0678509	0.0443554	0	0.0649347
Meox2	23.0984	8.37563	2.57519	4.58155	0	0.0424645	0.0206658
Pitx1	0.245572	176.955	40.5515	25.9238	1.91733	0.0989473	0.258147
Pitx2	11.0386	3.51832	15.5094	12.1844	18.108	15.5345	10.7844
Pitx3	0.656707	0	0	0.048101	0	0.089463	0.94361
Myf5	3.60262	0.358569	0.0903898	0.143461	0	0	0.0279389
Myf6	0.0504796	0.0437275	0	0	0	0	0
Dlx1	2.0803	2.03759	0.347723	0.211478	0.119191	0.107677	0.308071
Dlx2	7.63434	7.54549	4.31935	1.64669	0.191472	0.192636	0.410593
**Dlx5**	**4.8294**	**5.30261**	**5.40332**	**5.49241**	**0**	**0.042063**	**0.0448527**
Lbx1	19.4047	14.3668	0.588205	0.647748	1.36827	0.20259	0.266896
Lbx2	0.176251	0.317431	0.0881365	0.257786	0	0.160073	0.0850159
Evx1	4.40766	1.87491	2.18454	0.309646	0.134535	0	0
Lmx1a	0.558351	0.739587	0.161246	0.259575	0.224278	0.051276	0.0165677
Lmx1b	58.8766	63.961	5.09754	3.99891	1.04315	1.11573	1.23868
**Jag1**	**6.44265**	**3.85672**	**5.45555**	**4.89323**	**2.111**	**3.54299**	**2.57341**
Jag2	10.7073	10.2832	8.7727	8.61176	11.4039	23.6076	28.8666
En1	2.74341	2.4147	1.14929	1.61345	0.612985	1.00419	0.659871
En2	0	0	1.53908	3.01124	0.668965	1.24968	0.970712
Mog	0.0694745	0.0920985	0.0732134	0.105346	0.163806	1.06696	0.949599

HOX transcription factors

Hoxa1	0.679508	0.897361	3.00463	4.45891	3.85168	4.34817	4.85121
**Hoxa2**	**3.73543**	**2.92742**	**4.52491**	**5.3456**	**0.255411**	**0.220216**	**0.225672**
**Hoxa3**	**10.3435**	**8.99015**	**5.86199**	**5.96029**	**1.06461**	**1.30226**	**0.488617**
Hoxa4	2.85292	2.09997	1.77263	2.01744	0.436238	0.569353	0.61906
Hoxa5	21.6693	17.7632	6.07767	6.80512	0.767485	0.913254	1.04486
Hoxa6	11.6316	8.75624	1.81836	0.619937	0	0	0
Hoxa7	22.456	15.0538	8.8659	7.88693	0.543855	0.471061	0.152793
Hoxa9	94.9514	77.0644	10.2438	6.6549	0.339014	0.503456	0.491208
Hoxa10	140.411	137.497	10.9074	8.53987	0.511009	0.269297	0.420438
Hoxa11	77.8928	79.6439	10.694	7.53685	0.207571	0.0229215	0.163433
Hoxa13	13.7286	6.13086	1.62043	1.00489	0.887693	0.242069	0.273141
Hoxb1	0.263823	0.163253	0.0757003	0.170217	0	0	0
**Hoxb2**	**10.1712**	**7.58988**	**8.84227**	**9.88551**	**1.14194**	**0.429984**	**0.635493**
**Hoxb3**	**15.5444**	**9.03791**	**10.0796**	**10.1138**	**1.14734**	**0.487927**	**0.190532**
Hoxb4	3.33696	1.85601	6.08123	7.17635	1.50274	0.633982	0.333289
Hoxb5	5.45048	3.08501	1.65578	1.5747	0.116106	0.259884	0.0592923
Hoxb6	7.44606	6.26255	2.59088	1.10071	0.127488	0.357321	0.409925
**Hoxb7**	**3.73702**	**7.29207**	**4.87969**	**2.61598**	**0.436472**	**0.585157**	**0.236978**
**Hoxb8**	**5.28756**	**9.72632**	**6.37805**	**2.35959**	**0.179695**	**1.07686**	**0.5397**

**Hoxb9**	**0.952611**	**12.9594**	**12.0957**	**3.50208**	**1.16114**	**3.02315**	**3.35469**
Hoxb13	0	0	1.61752	0.439049	1.70498	1.49739	2.03595
**Hoxc4**	**8.44955**	**5.57337**	**8.68496**	**6.71667**	**0.85438**	**0.364068**	**0.821254**
**Hoxc5**	**5.9824**	**2.0779**	**5.25836**	**4.67873**	**0.129342**	**0.202676**	**0.0545721**
**Hoxc6**	**15.0241**	**10.366**	**7.38581**	**7.28191**	**0.678214**	**1.27896**	**0.983493**
**Hoxc8**	**2.91471**	**5.31873**	**3.53824**	**1.73341**	**0.246709**	**0.633316**	**0.630068**
Hoxc9	1.59283	11.781	2.02833	1.4487	0.64896	0.0891928	0.250461
Hoxc10	0.355147	47.8128	2.51934	1.1257	0.225081	0.464997	0.492305
Hoxc11	0.136082	23.9723	0.338671	0.0819786	0	0.183168	0.0806175
Hoxc12	0.14174	0.560111	0.282828	0.2064	0	0	0.0688265
Hoxc13	0.505071	0.36348	1.85988	2.32218	1.5659	0.958146	1.33314
Hoxd1	0.623704	0.928866	1.76617	1.11531	0.23532	0.120449	0.0308291
Hoxd3	4.63632	4.02328	2.72668	2.93906	0.0771168	0.0952723	0.0351857
Hoxd4	25.6768	15.8781	4.95451	2.59976	0.308445	0.277161	0.497798
Hoxd8	30.634	17.9393	8.52023	6.88076	1.44745	0.634578	0.56248
Hoxd9	94.0257	49.6073	14.0134	9.38877	1.15558	0.496137	0.318454
Hoxd10	61.3826	39.345	6.44433	3.25347	0	0	0.0278281
Hoxd11	165.499	78.6488	6.46441	3.41626	0.287081	0.184503	0
Hoxd12	33.7959	15.9576	2.72343	1.02592	0	0.0212247	0.0213227
Hoxd13	56.2849	23.4679	3.57753	1.42614	0.228505	0.432572	0.293781
				T-box genes			
Tbx2	18.0698	14.4142	48.8044	44.8117	0.646942	0.292124	0.401673
Tbx3	21.2613	16.6262	34.979	26.3951	20.7587	13.0461	19.362
Tbx4	4.00826	45.0306	0.136731	0.217436	1.63203	1.48997	1.16517
Tbx5	44.9254	0.249299	0.998799	0.747425	0.0879211	0.223256	0.111104
Tbx6	0.569514	0.309853	1.09221	0.96071	0.691704	0.681308	0.616006
Tbx15	129.355	77.2259	5.30537	12.8616	1.25817	2.08379	0.985277
Tbx18	28.0709	13.4202	5.79198	5.9929	0.541288	0.349802	0.210402
Tbx19	0	0	0.0218001	0.0209732	0	0.0407884	0.020416
Tbx20	0.0664201	0.220307	0.323426	0.338021	1.86421	0.184126	0.216497
Tbx22	0	0	0	0	0	0	0

Supplementary Table 3. Expression values of 279 genes identified by Taher et al. **(**Taher L, Collette NM, Murugesh D, Maxwell E, Ovcharenko I, Loots GG. Global Gene Expression Analysis of Murine Limb Development. Emanueli C, editor. PLoS ONE. 2011 Dec 9;6(12):e28358–60**)** that are suggested to be involved in limb development. Genes with FPKM <5 in all samples were not considered. Genes that are differentially expressed between control and induced fibrin bodies are highlighted in green (fold change >=2 or <=0.25). Of these, genes that have the similar expression to the limb are highlighted in bold green (fold change between 0.25 to 2).

**Gene**	**Forelimb**	**Hindlimb**	**FB.1**	**FB.2**	**CTL**	**2C-iPSC.1**	**2C-iPSC.2**
1700066M21Rik	5.97507	5.87152	5.25946	5.32743	5.79957	4.4864	4.12554
**a**	**9.43463**	**6.81888**	**9.36484**	**6.32013**	**0**	**0.206183**	**0**
Aaas	49.9661	53.3592	56.2276	61.1966	72.1348	72.1402	79.7695
Ada	4.38971	4.64673	1.53959	1.45942	6.25102	2.62413	2.37858
**Add3**	**21.4811**	**20.06**	**31.0417**	**31.5029**	**15.5207**	**19.9818**	**17.0992**
Aes	147.987	121.818	100.316	101.213	72.1284	160.192	173.831
Akt1s1	71.614	91.7589	51.1884	45.9188	76.7708	31.723	159.726
Alx1	2.971	3.75122	2.59097	3.03621	0.0668391	0.176646	0.0212798
Alx3	22.5724	39.9693	2.03759	2.63218	0.0809824	0.0547531	0.141188
Arid5b	7.48484	4.36672	23.4639	18.678	14.5408	14.2779	12.9446
Arpc3	93.8783	108.653	124.422	144.497	120.776	123.282	137.229
Arpc4	136.865	119.486	105.721	111.77	132.357	80.0744	84.1887
Asb4	322.367	247.348	0.632676	0.563019	0.0998365	0.295113	0.31642
Asna1	72.2988	71.9801	86.8699	98.8152	91.2231	165.064	148.962
Aspn	0.0828421	0	0.0697353	0.0217843	0.431041	0.333857	0.465907
Atp11b	11.2822	11.5468	28.5715	25.7978	26.8905	45.4036	47.7695
Atp5j2	155.431	149.233	93.3196	107.906	102.787	151.647	127.179
Atp5l	268.24	229.602	223.942	250.108	208.341	219.503	206.315
BC005561	4.28396	4.63218	3.11478	2.8194	2.5028	3.83536	2.69022
Bcam	32.9739	34.3344	16.5027	16.1985	25.5172	60.5514	57.5049
Bhlha9	14.6683	11.4403	0	0	0.0997893	0.0508436	0
Bin1	43.4705	43.6431	19.616	21.3425	14.2129	6.51658	6.72576
**Bmp7**	**22.8271**	**21.7391**	**13.5691**	**14.0194**	**4.6323**	**5.93613**	**6.02421**
Btbd11	4.06324	4.5713	2.15612	2.55918	4.5385	4.62176	4.18827
Calml4	1.81467	1.48259	0.788217	0.557941	0.84301	2.93706	3.08841
Cap1	75.7587	80.8458	101.425	102.946	116.407	98.5496	104.79
Capg	11.1515	7.93868	15.7484	20.3426	47.7488	23.3383	22.5894
Capn6	292.975	270.91	12.2044	16.9799	15.6863	0.768553	1.71721
Casc5	11.0802	12.7698	7.28925	10.5543	9.8199	13.5003	13.4493
Cbl	18.1121	21.802	10.9715	9.46125	11.5981	17.3421	14.6277
Ccdc109b	3.69631	1.1261	21.2602	9.35552	0.574637	0.77312	0.771887
Ccdc39	1.80535	1.41836	1.75001	1.45397	1.14458	1.1799	1.46349
Ccnl1	28.5051	27.1427	36.7798	35.9407	25.6661	41.5737	41.038
Cdc23	59.4383	59.758	34.2542	34.6517	33.4441	40.1538	39.5293
Cdk14	7.64205	6.61778	2.18958	2.33163	3.59193	0.631908	0.276999
**Cdo1**	**49.5215**	**22.6112**	**47.2317**	**57.536**	**5.15347**	**4.38533**	**7.4874**
Cdx1	1.27655	2.15548	4.50776	3.05864	4.57149	0.829762	2.19127
Cfl1	1073.74	1015	519.853	564.002	484.187	600.782	668.461
Cldn6	22.8024	27.5802	140.691	165.633	91.2225	66.0886	71.8463
Clk4	23.3636	19.7715	27.5694	28.7608	19.0628	21.8142	24.1004
Col14a1	0.528986	1.68861	2.88546	3.60104	0.181499	0.362572	0.194285
Col1a1	27.9195	13.6334	211.606	259.195	44.5925	116.535	80.2768
Cox6b2	12.5458	11.0168	137.338	137.335	77.0423	114.486	118.561
Cpz	3.0661	2.89736	2.42868	1.54454	1.00615	1.4403	1.66936

Crebzf	37.9763	35.3851	28.2726	21.7202	21.7969	16.0375	18.5483
**Crlf1**	**61.5094**	**34.9428**	**25.297**	**32.2074**	**2.52325**	**6.28684**	**4.73448**
Csk	69.9063	65.4519	18.9822	19.0026	28.5358	22.74	24.4686
Csrp1	42.4359	44.5177	67.8167	79.0992	264.847	55.9406	71.8665
Ctse	0	0.0275353	0.0534536	0.0513184	0.0306952	0	0
Ctsk	3.28558	1.69028	6.90403	9.1033	3.12646	9.45835	5.84257
Cxcl14	22.5271	15.2572	5.57624	4.0933	2.60068	1.33491	1.73273
Cytl1	0.137514	0	0	0	0	0	0
Daam2	***21.4113***	***19.4244***	***17.871***	***11.9667***	***0.252755***	***0.247842***	***0.270719***
Dkk2	2.04847	0.995971	3.59318	4.40577	0.172665	0.361789	0.361559
Dlk2	0.332553	0.252177	2.96722	5.08517	2.05875	0.907293	0.423259
**Dnm1**	**38.0195**	**24.0078**	**26.0383**	**25.5237**	**5.33389**	**15.2887**	**17.8535**
Dnm3	2.08875	1.51086	1.19618	0.958451	0.223766	0.136586	0.0202819
Dusp7	61.6181	56.8596	16.8563	19.8997	16.7196	10.0533	10.6758
Dynlrb1	159.783	143.41	125.761	123.64	105.041	105.03	113.484
E130012A19Rik	57.2814	53.411	78.3965	75.6494	74.8338	54.4046	68.5891
**Efna4**	**38.8253**	**34.8108**	**18.3384**	**20.5926**	**7.54674**	**6.55617**	**7.98012**
Epha3	4.06023	2.08416	3.75059	4.66923	0.0365137	0.189093	0.0342185
Ephb4	75.8043	63.1137	26.7806	27.2575	20.1979	21.0805	21.243
Etv5	43.3547	46.7813	32.7895	41.4962	48.348	94.6367	77.9298
Fam171a1	23.1768	21.6653	9.52134	8.49551	3.89766	3.46954	2.63445
Fancl	9.60248	10.4213	5.36108	4.94982	7.94762	14.2445	10.8656
Fbn2	52.3076	54.1659	24.2069	24.7535	0.437992	0.582973	0.297882
Fcho2	13.0266	11.3865	23.9874	21.245	23.401	25.5913	28.1986
Fgf18	0.971816	0.419921	0.142451	0.0687277	0.125288	0.639095	0.514802
Fgf8	69.6968	75.7258	1.56978	0.303374	0.405132	1.91589	0.602125
Fibin	15.5004	4.95348	0.127302	0.387891	0.12301	0.268051	0.212481
**Figf**	**9.12008**	**5.67653**	**5.92072**	**8.90633**	**0.822256**	**2.71416**	**1.38565**
Frg1	41.1702	39.9226	32.4641	38.4829	43.5341	41.0104	45.8737
Fyttd1	34.1727	35.6663	39.1459	41.0539	56.546	28.9044	38.175
G6pd2	0	0	0.0242751	0	0	0	0
Gabrp	0.90219	0.0904544	0.231922	0.509741	0.275698	0.189927	0.314652
Gal	0.100843	0	0.408122	0.802563	0.18788	0.448887	0.945391
Gdf5	0.913211	0.236897	0.116368	0.0897735	0.151789	0.260373	0.284933
Gdpd5	4.21615	2.95209	13.9666	10.1197	13.4633	2.96043	3.04108
Gorab	11.179	9.77145	10.3964	10.0469	5.67528	6.81228	7.04244
Grem1	18.898	20.0018	11.8786	10.7486	5.75164	0.742682	0.619742
Gsc	27.0529	8.48962	0.456637	0.398774	1.23991	0.271938	0.894929
Hadha	41.1836	41.1351	26.9481	28.3446	27.7321	34.0971	33.2489
Hdac8	8.4672	7.68585	3.84209	3.94113	2.01169	2.66155	1.95316
Hipk3	12.8229	14.753	12.7169	10.8297	14.7598	7.72483	8.11292
Hist1h2ae	6.96533	4.28168	0.530833	0.7955	1.75806	10.9413	6.88122
Hist1h2bp	13.5549	13.6227	16.4225	14.9518	17.9727	29.2556	25.8943
Hoxa11	77.8928	79.6439	10.694	7.53685	0.207571	0.0229215	0.163433
Hoxa13	13.7286	6.13086	1.62043	1.00489	0.887693	0.242069	0.273141
Hoxa9	94.9514	77.0644	10.2438	6.6549	0.339014	0.503456	0.491208
Hoxb5	5.45048	3.08501	1.65578	1.5747	0.116106	0.259884	0.0592923
Hoxb6	7.44606	6.26255	2.59088	1.10071	0.127488	0.357321	0.409925
**Hoxc5**	**5.9824**	**2.0779**	**5.25836**	**4.67873**	**0.129342**	**0.202676**	**0.0545721**
Hoxd10	61.3826	39.345	6.44433	3.25347	0	0	0.0278281
Hoxd11	165.499	78.6488	6.46441	3.41626	0.287081	0.184503	0
Hoxd12	33.7959	15.9576	2.72343	1.02592	0	0.0212247	0.0213227
Hoxd13	56.2849	23.4679	3.57753	1.42614	0.228505	0.432572	0.293781
Hoxd8	30.634	17.9393	8.52023	6.88076	1.44745	0.634578	0.56248

Hoxd9	94.0257	49.6073	14.0134	9.38877	1.15558	0.496137	0.318454
Hyls1	10.2291	9.86905	7.34956	9.0167	10.2295	13.1329	17.686
Id1	131.751	107.508	288.805	269.808	17.5547	19.5787	14.8277
Ifitm2	312.482	319.71	315.586	349.642	249.82	639.19	623.999
Ifrd2	29.9204	36.5611	15.397	17.6033	18.4134	29.2732	25.7846
Igfbp5	44.2553	56.0146	11.2949	16.8814	9.98942	2.40143	1.73424
Kcne3	10.2677	6.90358	0.908163	0.966859	0.308421	0	0
Kctd12b	1.68331	2.67193	0.595182	0.457055	0.207955	0.15818	0.177665
Kdm5a	11.097	10.2874	14.6233	11.9621	15.5991	11.3464	13.8194
Kera	0.0591956	0	0	0	0	0	0
Khsrp	95.2811	100.232	53.4872	47.5024	60.4846	85.5298	94.7011
Krt14	3.80085	1.5651	1.82626	3.28905	30.2811	0.142687	0.0888338
Krt17	1.22492	0.290155	0.307395	0.264884	1.29796	1.57263	1.4102
Krt5	24.5311	18.848	0	0.331031	0.461283	0.0447305	0.0459119
Lad1	3.04634	3.81134	10.1207	7.60439	10.3645	3.92952	4.20434
Laptm4a	151.906	150.768	303.444	338.514	231.26	169.585	202.616
Lbx1	19.4047	14.3668	0.588205	0.647748	1.36827	0.20259	0.266896
**Lef1**	**30.8284**	**35.8057**	**37.831**	**31.3818**	**2.28727**	**9.06981**	**8.20697**
Lhx2	75.0331	90.8337	2.8698	2.34693	0.466974	1.35984	1.80761
Lhx9	86.4284	86.7674	1.98586	1.24511	0.0800597	0.0226796	0.0532941
Lix1	118.07	95.4479	8.13895	4.72153	0.341897	0.510563	0.299873
Loxl2	13.4397	9.647	77.661	84.6951	43.0369	48.021	35.2054
Lsm2	114.252	117.653	46.1501	49.8325	73.7525	84.1239	85.5093
Map2k6	35.7863	34.0546	10.1205	11.5148	10.709	10.7864	12.5845
Matn4	0	0	1.02865	0.419072	0.440676	0.224327	0.311113
Maz	201.68	186.627	92.9568	97.2584	97.9985	170.723	167.001
Megf6	23.0254	19.5722	3.65303	3.87921	5.63757	4.25543	4.14533
Met	9.25183	6.50032	3.96534	3.60957	3.00035	1.41688	1.40898
Mme	6.91662	7.7273	17.8207	20.5131	21.3684	11.7556	18.1392
Mmp2	115.017	90.0185	39.2394	39.6548	15.5644	25.9644	15.5644
Msx1	130.815	162.33	30.272	14.2444	0.416495	0.854331	0.774057
**Msx2**	**23.8193**	**14.6941**	**29.9828**	**16.4369**	**10.0444**	**0.545068**	**0.465049**
Mup1	0.107705	0	0	0	0	0	0
Mybpc1	2.76997	1.34003	13.0164	4.58809	0.113109	0.0432087	0.061784
Myl1	0.0924339	0.398657	0.191725	0.371841	0.420568	0	0
Net1	41.0093	38.6494	32.6418	31.9351	32.1068	20.1178	21.8142
Nnat	1049.51	968.215	85.6294	94.3452	13.296	19.2857	15.3542
Nog	0.438899	0.488936	6.6218	5.09084	8.74624	0.628629	0.501818
Nolc1	68.4859	78.5039	26.4518	28.8153	33.5616	39.4161	34.6194
Npm3	80.5701	95.89	55.1231	63.2277	87.7264	137.371	142.219
Nrk	8.2377	7.77015	7.58915	8.52963	77.8522	0.727019	1.0228
Nsa2	69.6815	74.1745	92.5636	98.4942	69.0341	55.7489	61.3349
Ntf5	2.63918	2.18949	1.84193	1.59152	3.94581	1.51919	1.12461
Ogn	0.135294	0	0.0212857	0.0401423	0.167775	0.70825	0.303806
Ogt	50.6666	45.4692	92.7187	82.5496	63.352	52.7729	40.9399
Osr1	16.1168	6.76643	1.65111	3.18937	0.0985779	0.346907	0.125905
**Osr2**	**17.935**	**4.25681**	**8.07799**	**5.77826**	**0.823002**	**1.38068**	**1.12835**
Otub1	102.167	97.0934	57.3496	62.5674	54.707	46.9973	51.2829
Parvb	24.5522	34.6666	32.5406	30.1675	32.7393	23.0216	27.0694
Pcgf1	12.5643	14.1156	11.204	13.6959	9.84559	16.2026	16.8044
Pddc1	24.6406	20.9583	28.9373	33.2118	34.3099	32.3441	30.4665
Pdgfa	29.1849	26.1193	23.1474	26.6573	43.5556	9.56658	12.9285
Pdia4	111.4	117.022	55.6112	58.0359	64.093	91.1645	84.7748
Pdk2	3.90265	3.48095	9.86946	9.94152	6.94993	9.29613	10.8025

Pecam1	14.3698	15.1912	4.9807	7.638	19.9265	31.8823	31.3898
Perp	19.6115	19.0193	22.3007	26.3247	37.4585	17.4326	21.7774
Pfn1	1141.78	1067.52	437.249	504.507	615.123	681.987	779.426
Phf6	50.3703	52.1371	13.2761	12.4681	4.09508	12.5415	12.7262
Pi4kb	16.5087	14.5618	16.7345	18.1497	14.4282	11.372	11.3623
Pkdcc	167.195	98.8161	25.2516	29.2708	6.74713	4.60558	2.75991
Pknox1	13.1542	11.6304	9.62391	10.0969	9.68333	9.36633	8.67968
Plekhj1	31.1159	33.8534	21.8845	23.7435	27.8443	24.3979	29.2864
Pmaip1	32.5489	6.4358	39.2891	46.539	37.022	42.4687	58.4664
Ppp3r1	32.65	36.5691	39.5835	37.926	30.4371	29.1812	31.3582
Prl2c2	0	0.0716021	0.270664	0.610804	62.3889	2.99613	2.19912
Prrx1	167.133	150.165	65.1521	59.7432	2.57528	2.91353	1.53869
**Prrx2**	**171.751**	**128.428**	**79.787**	**78.1328**	**3.02358**	**6.59158**	**6.20404**
Ptbp1	364.345	405.717	189.707	197.457	183.07	312.124	337.456
**Ptk7**	**75.1419**	**60.3827**	**114.529**	**99.6463**	**23.2673**	**57.6785**	**57.542**
Ptp4a3	17.4563	16.5201	10.1673	10.9783	17.522	27.9917	31.5803
Pttg1	34.78	24.7199	49.3894	68.2936	102.387	109.705	138.828
Rab4a	12.3622	13.2579	17.1727	17.7965	17.5865	18.9786	18.5052
Rab5c	99.7538	103.832	140.608	162.064	109.238	91.5909	96.9742
Rabl3	15.4955	15.7189	11.0757	12.0328	9.09944	7.82009	7.7855
Rac3	11.3725	10.2214	31.5458	28.2577	9.08567	8.01017	8.89274
Ramp2	13.8717	20.2047	18.8448	21.3138	22.5832	2.49367	2.60936
Ranbp6	11.4101	9.74298	8.1229	7.88274	4.42365	3.25313	2.61819
Rarb	5.85522	14.5257	1.46739	1.94018	0.541896	0.325749	0.28498
Rarg	81.1608	61.1086	28.0062	23.033	17.7308	46.5494	48.4542
Rbm39	157.165	153.084	146.328	141.062	130.358	159.04	185.656
Rnf25	23.1687	20.6531	20.7198	19.518	23.1002	17.2147	16.009
Rps24	3736.78	4469.52	3919.38	4628.68	3113.73	3444.03	4413.21
Rspo2	5.86554	7.67423	0.0697683	0.0331241	0.115161	0.0166029	0.0161632
Rspo4	26.3424	17.6002	1.32825	0.763647	0.313664	0.820544	0.950447
**Rtl1**	**0.555663**	**0.771419**	**0.999117**	**0.947673**	**0.464853**	**0.437189**	**0.29622**
Scaper	2.40238	2.29482	3.92491	4.49655	5.38275	4.7712	4.43583
Scn2b	8.02517	9.29223	0.166405	0.234257	0.0868045	0.246523	0.145452
Scn4b	1.6593	3.86095	0.0221241	0.0212426	0.0318111	0.146413	0.14664
Sdccag3	30.0177	30.6953	24.0761	28.5662	27.5963	28.5117	28.6794
Sertad4	54.7685	49.403	7.68698	13.0174	1.45951	2.12292	1.88153
Shox2	134.374	77.5964	7.82733	12.0778	0.413723	0.249948	0.229572
Slc25a24	19.2488	17.8159	18.6635	17.0866	14.2915	2.67803	2.25202
Slc4a1	18.2199	24.8866	0.050299	0.0484703	0.130716	0.273527	0.202658
**Slc4a7**	**13.7914**	**15.835**	**19.1185**	**16.7464**	**8.84776**	**15.286**	**14.1068**
Smn1	54.3485	61.0439	34.7162	35.8582	35.8887	43.7921	51.165
Snai1	63.9455	61.1623	58.0177	51.8893	42.6552	4.87159	7.43761
**Snap91**	**2.67777**	**1.38631**	**3.32548**	**2.76875**	**1.30382**	**0.394736**	**0.564272**
Snrpb2	61.2918	77.8938	31.2503	32.9216	31.2448	30.3222	37.3585
Sox8	5.0172	8.41812	0.171377	0.714158	0.265453	1.43261	1.30099
Sp4	7.97996	6.37753	4.0627	4.1989	3.58967	4.36949	4.15837
**Spry1**	**29.3556**	**30.4344**	**23.6938**	**23.4322**	**4.53007**	**11.5913**	**11.7109**
Supt16	98.5511	106.214	57.3224	60.2561	62.711	100.321	94.5392
Taf7	9.72104	10.6942	36.1016	37.7648	42.4839	31.9143	31.1315
Tbx15	129.355	77.2259	5.30537	12.8616	1.25817	2.08379	0.985277
Tbx18	28.0709	13.4202	5.79198	5.9929	0.541288	0.349802	0.210402
Tbx2	18.0698	14.4142	48.8044	44.8117	0.646942	0.292124	0.401673
Tbx3	21.2613	16.6262	34.979	26.3951	20.7587	13.0461	19.362
Tbx4	4.00826	45.0306	0.136731	0.217436	1.63203	1.48997	1.16517

Tbx5	44.9254	0.249299	0.998799	0.747425	0.0879211	0.223256	0.111104
Tgfbr3	4.80494	4.24422	19.6985	14.4288	18.6098	3.74457	2.62582
Thoc2	22.6652	20.4258	34.2216	34.0247	24.1078	18.4357	19.6459
Tmed2	157.299	172.185	197.621	198.843	197.021	90.1308	105.869
Tmem119	73.3167	55.3614	11.5096	13.3604	0.76658	0.423083	0.404105
Tmem173	11.4076	13.911	1.33304	2.0005	0.550244	0.568308	0.306472
Tmem87a	13.0253	11.519	27.1928	30.1553	29.3963	21.4309	24.3282
Tnrc18	64.0181	50.8473	41.9293	33.8477	23.6502	12.5882	14.2585
Trp53	172.294	186.254	63.6498	63.7133	88.9889	142.791	142.018
Trp63	9.68747	9.96306	0.16922	0.207069	0.0935972	0.121726	0.0111572
**Tshz1**	**20.0539**	**18.4224**	**10.7385**	**8.6238**	**2.03582**	**0.533155**	**0.533435**
Tshz2	12.6174	13.2845	1.68838	1.72904	0.131484	0.251087	0.289495
Tshz3	4.78007	2.9178	8.76472	8.89352	0.553751	0.703271	0.425478
Ttc14	16.4863	16.446	22.6582	21.5098	20.0032	20.9742	18.7207
**Twist1**	**235.245**	**246.84**	**240.8**	**223.845**	**4.56031**	**7.11627**	**7.19662**
**Twist2**	**47.1151**	**45.7171**	**40.5805**	**34.2956**	**4.08041**	**4.48405**	**2.7415**
Ube2s	366.203	375.188	138.788	150.52	185.931	254.494	244.956
Ubl7	45.1915	40.9185	22.606	22.4136	37.7613	32.6835	33.2821
Upf1	57.3142	57.6866	21.4578	20.1607	37.9113	54.9293	59.5071
Usf1	47.606	44.8313	31.1101	31.441	29.9689	46.9824	44.7003
Vps72	103.887	95.497	37.6948	37.4341	38.0612	43.329	49.0723
Wdfy1	1.9866	2.05345	19.569	21.9559	15.2694	14.4714	15.0531
Wif1	*9.70041*	*5.06539*	*115.787*	*58.8053*	*0.0960344*	*0.0510613*	*0.0950714*
Wnt5a	55.9313	40.1678	14.6161	13.3926	1.67552	0.795035	0.55928
Wnt6	26.0981	20.5897	3.02657	2.30985	0.681776	0.532431	0.594223
Wnt6	26.0981	20.5897	3.02657	2.30985	0.681776	0.532431	0.594223
Wnt7a	4.90943	4.78793	0.313289	0.265261	0.840938	0.121928	0.209446
Xab2	33.6736	33.6165	29.5454	31.9151	43.8305	49.4707	51.0135
Zbtb12	65.0078	70.9933	14.5347	14.3709	7.04446	14.8785	14.1189
Zfhx4	22.8036	12.9883	5.47919	5.16916	0.19863	0.343089	0.251946
Zfp101	19.9099	21.2336	5.03554	5.77193	4.67429	5.91502	5.07291
Zfp146	60.4194	61.496	31.9391	28.5012	19.3513	17.0245	15.7904
Zfp2	5.92276	4.70699	3.90798	4.4919	2.74094	4.62313	5.01945
Zfp458	0.699602	0.647974	0.746991	0.474715	0.395859	0.189757	0.128882

Supplementary Table 4. PCR primers used in this work

Gene	Forward Primer	Reverse Primer	Product (bp)
Col2a1	CATCTTGCCGCATCTGTGTG	TGCCCCTTTGGCCCTAATTT	163
Gata4	CACCCCAATCTCGATATGTTTGA	GCACAGGTAGTGTCCCGTC	162
Gapdh	TGTGAACGGATTTGGCCGTA	ACTGTGCCGTTGAATTTGCC	159
Gli3	CATCCACCCTGCTCCAACAT	CTGTGAGGACTCAGAAGGGC	103
Kdr	GGATAACCTGGCTGACCCG	GAACCACAGAGCGACAGCTA	230
Meox1	CCGGACTGAGCGAATCTTCA	CTTCTCCGTCTCATTGGCGA	214
Nanog	TTGCTTACAAGGGTCTGCTACT	ACTGGTAGAAGAATCAGGGCT	100
Oct4	AGGTGAGCCGTCTTTCCAC	GGTCCACAGTATGCCATCCC	269
OC	CAGAGGGGAAGGGACAACAC	GGCTTCCTGCCAGTACCTTT	122
OPN	TCCCTCGATGTCATCCCTGT	ATCACATCCGACTGATCGGC	167
Pitx1	AGCACTCGTCGTTTGGCTAT	CAAAACCAACCTGGAGGCGG	215
Prx1	CAAGTTCCGCAGGAATGAGC	TGACCATGGCGCTGTACG	170
Runx2	GCGCATTCCTCATCCCAGTA	CTGGCTCAGATAGGAGGGGT	178
Sox2	AGTGGTACGTTAGGCGCTTC	CCCAGCAAGAACCCTTTCCT	207
Sox9	TCCCCGCAACAGATCTCCTA	AGGTGGAGTAGAGCCCTGAG	157
T	GGTATTCCCAATGGGGGTGG	GACCGGTGGTTCCTTAGAGC	142
Tbx5	AATCCCCAGCACAAACTCCA	GCGAGGTTCTATTCTCGCTCTG	125
